# Tailoring a brief intervention for illicit drug use and alcohol use in Irish methadone maintained opiate dependent patients: a qualitative process

**DOI:** 10.1186/s12888-016-1082-4

**Published:** 2016-11-03

**Authors:** Catherine Darker, Brion Sweeney, Eamon Keenan, Lucy Whiston, Rolande Anderson, Joe Barry

**Affiliations:** 1 Public Health & Primary Care, Institute of Population Health, Trinity College Dublin Russell Centre, Tallaght Cross, Dublin, D24 DH 74 Ireland; 2 Tara Trust, 12 Marchhall Crescent, Edinburgh, Soctland EH16 5HL UK; 3 National Drug Treatment Centre, McCarthy Centre, 30-31 Pearse St, Dublin 2, Ireland; 4 Suite 33, Morrison Chambers, 32, Nassau Street, Dublin 2, Ireland

**Keywords:** Alcohol, Illicit substances, Methadone, Opioid dependent, Brief intervention, Tailored, Qualitative, Focus group, Semi-structured interview, Randomised controlled trial

## Abstract

**Background:**

The World Health Organization (WHO) recommend the tailoring of a brief intervention (BI) programme of research to ensure that it is both culturally and contextually appropriate for the country and the environment in which it is being tested. The majority of BI research has been conducted with non-opioid dependent participants. The current study developed a tailored BI for illicit drug use and alcohol use to a methadone maintained opioid dependent polydrug using cohort of patients.

**Methods:**

Focus groups with staff and one-to-one qualitative interviews with patients guided the tailoring of all intervention materials for use in a subsequent cluster randomised controlled trial (RCT). This was done to make them contextually appropriate to an opioid dependent cohort and culturally appropriate to Ireland. Thematic analyses were utilised.

**Results:**

The BI was modified to ensure its compatibility with the culture of an Irish drug using population, with elements of motivational interviewing (MI) and personalised feedback incorporated. Example scripts of a screening and BI were included, as was an algorithm to facilitate clinicians during a session. Modifications to the ‘Substance Use Risk’ cards included weighting the severity of the problems, writing the language in the first person to personalise the feedback and including tick boxes so as to further highlight the relevant risk factors for individual patients. Photographs of key risk factors were included to display pictorially risks for illiterate or semi-literate patients. Examples of the interaction of particular substances with methadone were of particular importance to this group. Modifications of the ‘Pros and Cons of Substance Use/Reasons to Quit or Cut Down’ included additional categories such as addiction, crime and money that were salient to this cohort. The manual was used to standardise training across trial sites.

**Conclusion:**

The research team was faithful to WHO recommendations to tailor BI programmes that are culturally and contextually appropriate to the treatment cohort and clinical environment. Outcome data from the cluster RCT have demonstrated that the tailored intervention was effective.

**Electronic supplementary material:**

The online version of this article (doi:10.1186/s12888-016-1082-4) contains supplementary material, which is available to authorized users.

## Background

The World Health Organization (WHO) recommends a brief intervention (BI) as an intervention to address patients’ problematic substance use [1]. This is achieved through a two-step process. Firstly, the substance to be targeted in the BI is identified using the valid and reliable [2, 3] “Alcohol, Smoking and Substance Involvement Screening Test” (ASSIST). Secondly, the clinician delivers a BI for the most problematic substance, determined by the ASSIST. This involves helping patients to identify substance use problems, understand the risks involved and motivate a reduction or abstinence from substance use as appropriate [1, 2, 4]. BIs are underpinned by a theoretical framework drawing primarily on; i) principles of the Stages of Change model [5] identifying patients’ level of motivation to change, and ii) Miller and Rollnicks’ motivational interviewing (MI) eliciting change talk and moving patients through the stages of change [6], incorporating the FRAMES technique (Feedback, Responsibility, Advice, Menu of Options, Empathy, Self-efficacy).

The association between BIs and reduced alcohol consumption has been depicted in numerous studies [7–10]. The effectiveness of screening and BI on illicit drug use was examined with a sample of over seven hundred patients recruited from primary care settings in Australia, Brazil, India and the United States. After receiving a BI, 82.8 % of the sample attempted to reduce their illicit drug use, with 60.2 % succeeding in maintaining this reduction at 3 month follow-up [11]. To date, the impact of BIs on a variety of illicit substances has been examined and shown to be effective in relation to amphetamines [12], benzodiazepines [13], cannabis [14–16], cocaine [17, 18], and illicit substances in general [2, 11, 19].

However, the majority of studies of the effectiveness of BIs have been completed with non-opioid dependent patient cohorts who represent members of the general public attending primary care services, A&E departments or community mental health services. A single previous study, conducted by the current authors, has found evidence for the effectiveness of a WHO BI, within community methadone clinics [20]. At 3 month follow-up opioid dependent patients significantly reduced their alcohol consumption as a result of receiving a BI. Existing evidence, including our previous work, demonstrates the effectiveness of BIs in reducing alcohol consumption. Following on from this, a cluster randomised controlled trial (RCT) was designed to examine the effectiveness of BIs for illicit drugs and alcohol in Irish methadone maintenance clinics. Results of the cluster RCT have been published elsewhere [21].

Organisational culture and climate can have a profound influence on organisational functioning and effectiveness [22], and this in turn may influence the successful outcome of implementing an intervention according to the Quality Implementation Framework [23]. It has also been recognised that interventions have ‘core components’ (the essential and indispensible elements of the intervention) and an ‘adaptable periphery’ (adaptable elements and structures related to the intervention and organisation in which it is being implemented) [24]. In recent years, the WHO have advised that it is important to tailor BI programmes to the population at hand and to consider the context and culture of the service in which programmes will be delivered [11]. The UK Medical Research Council Framework for Developing Complex Interventions suggests specific phases to the development of health service interventions, such as, for example, systematically developing core components of the intervention [25]. Integrating qualitative research in the design of a complex intervention can contribute to optimising the robustness of intervention materials [26] and has been utilised within a number of complex interventions in the pre-trial phase of the design [27–29]. A recent study examining a brief motivational intervention for heavy alcohol use in dental practice settings conducted web-surveys and informal interviews with practitioners in the design of intervention materials that were used in a subsequent trial [30]. Qualitative focus groups were used in recent studies designing a risk reduction intervention for injecting drug users [31] and tailoring the treatment of depression in older adults [32]. Qualitative research, as well as providing important insights into processes of change, is a good way to involve users by allowing for a wider range of views to be canvassed and systematically incorporated into the intervention [25].

The degree to which an intervention can be adapted, tailored, and refined to meet local needs is a core component of implementation [24]. Therefore, the purpose of this paper is to describe the development and the process of tailoring of a screening linked BI, through qualitative methods. Intervention materials guided the standardised training of clinicians across all trial sites for the subsequent RCT.

## Methods

The methods of this research are reported utilising the consolidated criteria for reporting qualitative studies (COREQ) [33].

### Design

Focus groups (FGs) with clinical staff (*n* = 15) and one-to-one interviews with patients (*n* = 10) were utilised. FGs were chosen as the format best suited for the clinical staff as participants could build on each other’s ideas, through facilitated discussion in a group setting [34]. Also, FGs were chosen for pragmatic reasons to minimise disruption to the clinic by only requiring access to specific clinical teams once, thus reducing researchers’ time on the clinical floors. A one-to-one format was chosen for the patients in order to provide privacy and confidentiality. A semi-structured interview guide was utilised in both formats of qualitative inquiry. It employed open-ended questions grouped around particular themes of interest to the research question but also allowed participants the freedom to discuss what was important or pertinent to them, encouraging a free flow of conversation [35]. All patient and clinician participants were provided with copies of the draft intervention manual and material.

The Research Ethics Committee of the Drug Treatment Centre Board approved this study.

### Participants

Patient participants were selected using purposive sampling, which is designed to identify specific groups of people who possess characteristics relevant to the phenomenon being studied [36]. Patients were opioid dependent methadone maintained polydrug-misusing patients, not attending any of the study sites to avoid contamination. Patients were recruited through a forum representing service users of addiction services. They were approached by the patient organisation and then introduced to the researcher (LW) once they agreed to take part.

Clinician participants were chosen to represent the clinical teams (multidisciplinary teams comprising doctors, nurses, counsellors, outreach workers and pharmacists) that had already been randomly selected to the intervention arm of the cluster RCT. Clinicians were invited to participate in a focus group after a presentation of the research was made to the multidisciplinary team.

A total of ten semi-structured interviews occurred with patients (10 in total) and two FGs with clinical staff (15 in total). All participants that were approached agreed to take part in the research.

### Settings

The FGs with clinical staff took place in two large Addiction Treatment Centres in Dublin, Ireland. Approximately 90 % of all Irish persons on methadone programmes are resident in Dublin and receive their treatment there. The semi-structured interviews with patients took place in a neutral setting with an organisation that hosts a service users’ forum for patients attending for Addiction Treatment in the North Inner City of Dublin, Ireland. The facilitators of the focus groups are academics and not clinicians themselves and were not working in the services in which the qualitative enquiry was undertaken. Only researchers and participants were present in the room during the FGs and interviews.

### Procedures

All participants were provided with a participant information sheet about the research including the members of the research team and were given an opportunity to raise any questions. Confidentiality was assured. All participants signed a consent form. Both the FGs and semi-structured interviews were tape-recorded and took place between December 2012 and February 2013. On average the FGs lasted 45 min and the semi-structured interviews lasted 35 min. Authors (CD and LW) facilitated the FGs and interviews respectively. Both authors were experienced in conducting qualitative research with PhD and MSc qualifications respectively. Both were working in the Department of Public Health and Primary Care, TCD in academic roles. Participants were not provided with incentives to take part and were not known to the authors conducting data collection. Participants were told where the authors conducting the data collection were working. Both clinicians and patients were presented with a draft of the intervention manual and all related draft materials for comment. The intervention manual included a description and overview of the main components of the ASSIST screening tool and brief interventions such as motivational interviewing. A step-by-step guide was included with resources to be employed during a BI such as example pros and cons to change of substance use behaviour. This was to be used during the intervention by the clinician, not the patient. Draft materials provided to participants included examples of pros and cons for changing substance use behaviour, take home material and readiness to change rulers. There was some cross over between questions asked within the FGs and interviews, with both stakeholder groups asking questions that related to the substance risk cards, the pros and cons of alcohol and illicit drug use, and take home materials (see Additional file 1 for sample FG and patient interview guides respectively). These guides were pilot tested before use. Field notes were made after the FGs and interviews took place. Data collection continued until saturation was reached. This is where no new ideas or themes emerge during data collection.

### Data analysis

The recordings of both FGs and all semi-structured interviews were transcribed verbatim. To enhance rigour, a summary of the main points was given at the end of both FGs and interviews whereby participants were asked if it was an accurate portrayal of what had been discussed. This form of ‘member check’ allows for participants to confirm or correct any errors in the topics discussed [37]. Transcripts were not returned to participants. Two authors (CD & LW) independently analysed the data and no software package was utilised. Each transcript was examined in detail. Rigorous line-by-line coding was applied, with a focus on experiential claims and concerns [38].

Thematic analysis was chosen as the type of analysis that would be undertaken as the aim of the preparatory phase of the trial was to gather feedback from clinicians and patients on drafted intervention materials with a view to making changes to materials ahead of the training and start of the trial. Themes were not predetermined in advance but rather they emerged from the data. Patterns in the data were then clustered into a thematic structure. Thematic analysis was utilised to identify and categorise major themes, sub-theme concepts and categories [39]. A thematic analysis was chosen as the qualitative analytical method due to its realist and constructionist perspective [40]. Thematic analysis is a flexible and useful research tool, which provides a rich and detailed, yet complex, account of the data [41]. Thematic analysis involves the search for and identification of common threads that extend across an entire interview or set of interviews [42]. The themes were then reviewed and refined to ensure they formed a coherent pattern of major and sub-themes and were recoded if necessary. Any differences in interpretation by the researchers were resolved through discussion. An inter-rater reliability of Cohen’s Kappa of 0.85 demonstrated a high level of agreement between the two raters. This form of multiple coding can enhance the rigour of qualitative analysis [43]. In reporting the results, the identities of the participants were anonymised by providing them with a pseudonym. For clarity in the quotes participants who were patients were referred to as ‘Mary, patient’ and participants who were members of the clinical team are referred to as ‘Sean, clinician’.

## Results

The results will focus on the tailored elements of the BI that were modified based on feedback from clinicians and patients through focus groups and interviews. (See Additional file 2 for copy of the tailored BI manual).

### Structure & components of a BI manual

The WHO describes the ASSIST and a BI across two manuals; in the current study both elements were incorporated into one manual. Balanced by the necessity of having enough information the decision to streamline the manual was taken because of the research team’s tacit knowledge of the busy, resource constrained clinical environment within which the clinicians work.

Participants suggested changes to the style of the manual. For example, the research team had included a single page ‘step by step’ algorithm guide to a BI to be displayed on treatment room walls. Participants in general found the drafted algorithm helpful; they did suggest alterations to its presentation, which were incorporated into the final version. An algorithm was developed for the tailored manual which incorporated the ASSIST and the BI together diagrammatically with the use of timing guidelines, the ‘5 A’s’ (**A**sk, **A**dvise, **A**ssess, **A**ssist, and **A**rrange) and colour coding for the ASSIST.

Peter, clinician: *I also really like the algorithm but I think it needs to be descending*, *that would make it much easier to read. And maybe if you put the timings beside it*, *to give us an idea of how long we are meant to be spending on each section*.

A sample script of a screening and BI session, reflective of a session between a clinician and a patient within an Addiction Treatment Centre, was developed. For the purposes of training, the sample script, the role-play and the algorithm were synergised so that they reflected one another for clarity purposes in training.

Patricia, clinician: *The example script is very helpful*…*You could nearly hear a session happening*.

### Substance risk cards

Both clinical staff and patients were asked for their opinion on the draft Substance Risk Cards. A Substance Risk Card was created for each individual substance that was assessed within the ASSIST screening tool. These cards serve the purpose of outlining particular risks that are associated with the use of specific substances. The use and purpose of the Substance Risk Cards were the same as envisioned by the WHO. However, in order to make them culturally and contextually appropriate, we made a number of changes based on feedback from the FGs and interviews.

### Tailoring of substance risk impact

For example, health and psychosocial problems were organised into separate categories of problems. An attempt was made to weight the problems in the categories with the more problematic risks at the top. We included how the particular substances might interact with methadone and exacerbate problems for this cohort in particular. The language on the Substance Risk Cards was written in the first person. ‘Tick boxes’ were included, as suggested by clinicians, so that during a session with a patient the clinician could tick the box relating to a set of risk factors that were pertinent to that particular patient and therefore tailor the BI to the patient based upon their individual risk profile. Photographs of key risks were included in order to display risks pictorially for illiterate or semi-literate patients.

Mark, clinician: *Sometimes you may just talk to them* [*patients*] *about their liver risks or whatever but that may not all sink in if you*’*ve a big long list like this it*’*s very detailed*, *so maybe if you can break them into categories with space* …*Tick the ones that are relevant to them*.

### Modification and simplification of language

Participants in the FGs made suggestions for simplifying the language and reordering the presentation of the risks associated with particular illicit drugs and alcohol.

During the interviews with patients there were some in vivo examples of patients not understanding some terminology.

Frank, patient: *What*’*s this one mean*? *Increased risk of* ‘*psychosis*’, *how do you mean*? *I don*’*t understand that word*.

### Literacy concerns and pictorial solutions

Clinicians expressed concern that there may be too much detail on the Substance Risk Cards and that pictures should be considered to represent some of the risk factors. This may also help with patients that have literacy problems.

Paula, clinician: *Pictures. Explanatory pictures. Just thinking about literacy as Mary was saying earlier*, *like the literacy sort of skills that individuals have might be quite poor*.

### Imagery to underline risks

Patients also spoke about the potential use of powerful imagery to underline the risks involved in drug use behaviours.

Stephanie, patient: *I think its good to shock people about the reality of using drugs*, *the risks involved*, *that would probably hit home. If there was an ad where two people bought gear* [*heroin*], *and they went to a flat*, *took it and then they feel asleep and one guy wakes up and his friend is dead. Something like that to kind of hit home. Like you need to include really shocking pictures*.

### Interaction with methadone and importance of context specific examples

A particular modification of the Substance Risk Card was the inclusion of the interaction between methadone and the target substance. This is a concern for this cohort of patients in particular given that here may be possible compromised liver function due to Hepatitis C in patients on methadone treatment.

Noel, clinician: *That*’*s* [alcohol and methadone impact] *important. I would put that interaction at the top of the page. You really need to do that with all of the substances*.

Both patients and clinicians spoke about the importance of context specific examples relating to the cohort of methadone patients and suggested modifications to the existing Substance Risk Card template. Clinicians in the FGs questioned the hierarchy of problems that patients are concerned with and suggested that patients are less concerned with physical health risks but rather psychosocial risks.

Mark, clinician: ‘*High blood pressure*’-*they don*’*t really care about that kind of stuff*…*whereas if you say*, ‘*a relationship problem with your family*’ *then that would have more meaning*…*I know the other stuff*’*s important but in their lives but there*’*ll be some things that will be of more relevance to them than others. I think psychosocial problems are very important. They are the biggest problem*.

Patients did however recognise physical risks as a consequence of drug taking, such as dental damage and a general detrimental effect of long-term drug use on appearance.

Anita, patient: *The dental damage*, *appearance and all*… *Let people know*, *like you don*’*t realise how much your appearance has gone down*, *yeah. My teeth are in an awful state*.

### Criminal behaviour

A patient indicated that criminal behaviour is particularly associated with this cohort, especially in relation to intravenous drug users and that this risk needed to be highlighted within the Substance Risk Cards.

Ronan, patient: *I shoplift because it*’*s so easy. I just go in and hold up a place with a shotgun. My sister comes with me. She goes in*, *folds up the clothes and puts them in a bag*” …*Nothing matters once you get your fix into you. You don*’*t care about the consequences*.

### Pros and Cons of substance use/reasons to quit or cut down

The “Pros and Cons of Substance Use” element of a BI is used by the clinician to help patients examine the reasons for the use of the target substance in the first place and to consider the reasons to quit or cut down on the target substance. These are considered prompts for clinicians to draw from. The patient’s own suggestions and reasons are the primary focus within a BI session. The “Pros and Cons” section was used in the same manner as outlined by the WHO. However, a number of changes were made based on feedback from clinicians and patients so as to make it contextually and culturally appropriate to our cohort. For example, we provided a lot more detail in the examples and also additional categories were created such as ‘addiction; ‘crime’, ‘money’ that would be particularly salient to our cohort.

### Reasons for drinking alcohol or using illicit drugs

Patients gave their views on the drafted “Reasons for Drinking or Using Drugs”, many of which resonated with patients, as exemplified in the following extract:

Tina, patient: *Reasons for drinking or using drugs*. [*reads*] “*To get out of me head*”, “*to relax when I*’*m stressed*”; “*to cope with feelings of boredom*”. *Yeah I recognise myself in each of them. You don*’*t have the word* ‘*despair*’ *in there do you*? *You should also put in more about depression and loneliness*.

### Reasons to quit or cut down from alcohol or illicit drugs

Some clinicians were quite critical of the drafted Reasons to Quit or Cut Down from Alcohol or Illicit Drugs, and made further suggestions for improvements.

Ruth, clinician: *I don*’*t think the reasons for quitting or cutting down are great*-*they*’*re too vague*. “*To live longer and feel better*”, *I mean saying that to a 30 year old would be a waste of time*-[*reads*] “*consume fewer empty calories*”, *well that*’*s going to be of no interest for men*. [*reads*] “*To sleep better*”, *many of them* [*patients*] *may not even realise they*’*ve got sleep problems. The dangers of alcohol and drugs are so huge I just feel that list is not great*… “*to be a better parent*”, “*have a more normal life*”, *I*’*d actually be more straight forward and say* “*to prevent family breakdown and children being taken into care*” *because that*’*s actually something our cohort is seriously hit with*…*so really just go for the jugular*.

Financial Reasons to Quit or Cut Down also resonated with patients.

Nigel, patient: *Yeah*, *I think they*’*re all solid reasons to quit or cut down. Definitely to have more money. Like I mean as I was saying earlier I think I could probably have* [*laughs*] *a property portfolio*.

Two particular issues that are pertinent to this cohort within Reasons to Quit or Cut Down their substance use were criminal activity and subsequent court convictions.

Stephanie, patient: *If you have a charge or a* [*criminal*] *record*… *and you*’*re on the drug courts*, *some people will cut down*, *not because they want to but because it*’*ll look good for the courts. And also they want the kids back off the courts*.

### Take home materials

A discussion took place as to whether patients would value any Take Home materials from the BI session. It was proposed that Take Home materials would include two tailored pieces of information resulting from the BI; firstly, the Feedback Report Card with results from the ASSIST screening tool that would identify which of the substances that patients were using was most harmful; and secondly, the Substance Risk Card that would be personalised to the patient which would have each individual risk item ticked that was discussed during the BI. Discussion also took place as to how these Take Home materials would be presented to patients. There were mixed opinions expressed as to whether patients would retain the information from the BI that would be provided to them. When asked as to whether Take Home materials would be something that patients would value, a participant replied:

Amy, patient: *Do you know what an addict would do with that*? F**k it away when they get outside. No. They’*d just throw it away*, *they would*.

However, some patients thought that the Take Home materials would be of benefit, especially with a view to retaining the documents for future reading.

Nigel, patient: *I think if it was explained to me* … *if I was reminded every 2 to 3 months to take this out and have a look*…, *I suppose if it was in a little folder in terms of presentation and I could put it off to one side and come back to it every 2 to 3 months*.

Confidentiality was a key concern of both clinicians and patients with regard to how the Take Home material would be given to the patients. An innocuous folder with a neutral cover and title was suggested and subsequently adopted. This would serve the dual purpose of providing a reminder of the key risks involved with the continued use of the target substance, while also providing a generic folder that patients would be comfortable holding.

Tina, patient: *Put it in a folder. And I think it should be private*. … *Like* “*Healthy Living*” *or* “*Healthy Options*”, *do you know*, *something like that should be printed on the front*.

## Discussion

The present paper describes the process of tailoring a BI for use with opioid dependent methadone maintained patients. The intervention and accompanying manual was developed with feedback from clinicians and patients. Changes were suggested by participants and implemented if the majority of participants were in agreement with the change, if it was in keeping with the key components of BIs and if it was practical. Modifications included the ASSIST screening tool and BIs outlined in one manual, the incorporation of a one page step by step guide to a BI with timings and the 5A's algorithm. Also the tailoring of the sample script to a methadone maintained cohort, and the inclusion of cohort specific examples such as criminal activity and family breakdown within the Substance Risk Cards, use of pictures to help semi-literate and illiterate patients, and inclusion of interaction between particular substances and methadone.

Tailoring the BI in this way served a number of vital purposes. The manual was utilised in developing screening and BI training sessions that were delivered by an expert trainer (author RA). Training sessions were mapped directly onto the manual, which facilitated the standardisation of training sessions across trial sites. This allowed the research team to assess fidelity to the intervention manual throughout the training sessions. Treatment fidelity refers to the methodological strategies used to monitor and enhance the reliability and validity of behavioural interventions. Developing an intervention manual is considered a key step in reducing unintended variability in training sessions as outlined by the Behavioral Change Consortium [44].

There were a number of additional gains to devoting time during this pre-trial phase, such as increased stakeholder involvement, which led to better clinician buy-in for the trial itself. Ignoring the social context in which interventions take place has been identified as a significant barrier to translating evidence into on-going practice [45]. It has been recognised that intervention developers, the organisations in which the intervention takes place and staff therein, play a crucial role in the active implementation phase of an intervention [22]. This is known as the ‘ecological’ fit within the Quality Implementation Framework and is recognised as a key step in enhancing the intervention’s ‘fit’ to the clinical environment [23]. During the intervention design we also elicited feedback and input from a range of international experts in the area of BIs, which served to improve the quality of the final intervention. This allowed the intervention to retain the ‘core components’ of the original BI but allowed the researchers to adapt the ‘periphery’ elements [24]. For example, a core component of a BI is motivational interviewing, in which sessions are patient centred and directed which encourages the process of change by exploring ambivalence and facilitating patients to implement change [6]. This process was not altered in any way, as it is integral to a BI. Within motivational interviewing quite typically the Stages of Change ‘wheel’ can be presented to patients to assist in the determination of where in the process of change that individual patient sits [5]. We altered the language of pre-contemplation, contemplation, preparation, action, maintenance and relapse to be more understandable and in a plain English style to become ‘not thinking about it’, ‘thinking about it’, ‘getting ready’ ‘doing’, ‘minding’ and ‘falling back’. This would have maintained the core concept of the Stages but transformed the language to be more accessible to a population with low and in some cases no literacy. The investment in time and resources to undertake this qualitative process paid dividends for the subsequent trial. The results of the cluster RCT have demonstrated that a single clinician delivered BI can result in a reduction in substance use within a methadone maintained opiate dependent cohort, and this effect is sustained at 3 month follow-up [46].

Strengths of this study included eliciting feedback from both clinicians and patients and being faithful to recent WHO recommendations [1, 47] and those of the UK Medical Research Council when designing a complex intervention [25]. The purposive sampling techniques of stakeholder sampling and criterion sampling were chosen to select the clinicians and the patients respectively. Stakeholder sampling is particularly useful in the context of evaluation research and policy analysis. The findings reported here were based on qualitative analysis of FGs and interviews with a small number of participants. It is not the aim of qualitative research to achieve a representative sample in terms of either population or probability. Statistical representativeness is not a prime requirement, when the objective is to elicit opinions and feedback during the design phase of a complex intervention [36]. A potential limitation of the focus groups with clinicians was the possibility of introducing bias where clinical hierarchies may dominate the group discussion. However, the facilitators of the focus groups (CD and LW) were mindful of this and encouraged active participation of all staff members present regardless of clinical discipline or seniority. There is a strong identity within the opioid using community and the involvement of service users in this study has resulted in that culture informing the changes to the BI manual. The context within which persons used drugs and attended treatment services also is reflected in the periphery changes. Future research should aim to establish whether the tailored BI gained any added benefit beyond a generalised BI. Future research needs to develop a formal mechanism to assess fidelity of the delivery of a training session to the intervention manual. There is also a need to determine whether improvements in fidelity are made through the use of a carefully constructed training manual.

## Conclusions

There is a paucity of research that describes the process of tailoring interventions. It was feasible and helpful to use qualitative methods to identify necessary modifications to the BI that underpinned the subsequent trial. This approach generated a large number of suggested changes that were implemented ahead of commencing the intervention. The research team was faithful to WHO recommendations to tailor BI programmes that are culturally and contextually appropriate to the treatment cohort and clinical environment. Outcome data from the cluster RCT have demonstrated that the tailored intervention was effective [21].

## Additional files

Additional file 1: Clinician and Patient Interview Schedule. Intervention Development: Clinician Focus Group Interview Schedule and Intervention Development: Patient One-to-One Interview Schedule. Interview schedule outlines for data collection. (DOCX 88 kb)
Additional file 2: Screening Led Brief Intervention Training Manual. Screening Led Brief Intervention Reference Manual. Training manual developed as a part of this qualitative process. (DOCX 5115 kb)

## Abbreviations

‘5 A’s’: Ask, advise, assess, assist, and arrange
ASSIST: Alcohol, smoking and substance involvement screening test
BI: Brief intervention
FG: Focus group
FRAMES: Feedback, responsibility, advice, menu of options, empathy, self-efficacy
MI: Motivational interviewing
RCT: Randomised controlled trial
WHO: World health organization
