# Supplementary material for: Tailoring a brief intervention for illicit drug use and alcohol use in Irish methadone maintained opiate dependent patients: a qualitative process
Source: BMC Psychiatry. 2016 Nov 3;16:373. doi: 10.1186/s12888-016-1082-4 (PMC5094097; doi:10.1186/s12888-016-1082-4)
Supplement: Additional file 1: — Clinician and Patient Interview Schedule. Intervention Development: Clinician Focus Group Interview Schedule and Intervention Development: Patient One-to-One Interview Schedule. Interview schedule outlines for data collection. (DOCX 88 kb) [file 12888_2016_1082_MOESM1_ESM.docx]

**Appendix A: Intervention Development: Clinician Focus Group Interview Schedule**

1. **What do you think about the structure of the manual?**

- What do you think about how it was presented?

- What do you think about the 5 A's used to structure the manual?

- What do you think about the examples provided for questions?

- Do you think you would use it as a reference manual?

**2. What do you think about the substance risk cards? (p. 33-42)**

- Will patients understand the points made?

- Will they help patients to discuss their substance use?

- Can you think of any other problems we should include?

- What do you think about the sections on the overlap between methadone and illicit substance use?

**3. What do you think of the examples provided as prompts for the pros and cons of alcohol/drug use? (p. 44-45)**

- What do you think about the examples provided?

- Will they help patients to discuss their substance use?

- Can you think of any other examples we should include?

-How can we make it more Dublin?

**4. Do you think we should provide take home material?**

- Will take home material be used? *(Probe: literacy)*

- What do you think about giving the feedback report card as take home material? (p. 32)

- Do you think there providing the substance risk card to take home is a good idea? (p. 33-42)

- Is there a need to provide this information in a folder?

- Can you think of anything else that would be a good idea to give patients to take home with them?

That is all of our questions, is there anything that I have missed or anything that you would like to add?

Thank you for taking part.

**Appendix B: Intervention Development: Patient One-to-One Interview Schedule**

**Introductory Questions**

**We can get started now if you are comfortable with that …**

1. Could you tell me a little bit about your experience of methadone treatment?

(*Probe: where, how long, receiving at present*)

1. What are/have been your problem substances?

**Substance Risk Card**

- Explain what a Brief Intervention is.
- Show client a copy of an appropriate substance risk card.
- Explain how the substance risk card is used.

**1.** What do you think about the substance risk card?

*(Probe: Do you understand the points made? Do you think it would help you to understand the impact of your drug use/discuss your drug use?)*

**2.**What do you think about the impact examples provided?

*(Probe: Do the possible problems concern you? Can you think of any other problems that we should include? Which points motivate you? Which points do not motivate you?)*

**3.** What do you think about including picture?

*(Probe: Do you think this is necessary? What do you think about the examples provided?)*

**Pros and Cons of Drug/Alcohol Use**

**I would like to move on to the next section now. Part of the treatment is a discussion of the reasons for and against cutting down on substance use...**

- Show client a copy of the readiness/confidence ruler
- Explain how it would be used.

1. What do you think about the readiness/confidence ruler?
   1. *(Probe: helpful, understand, help you to talk about your substance use?*

Show client a copy of the pros and cons of use sheet

Explain how pro and con examples would be used.

1. What do you think about the examples on the sheet?
   1. *(Probe: Can you relate to the examples? Would they help you to discuss your substance use? Which examples motivate you/ do not motivate you?)*
2. Can you think of any other examples that we should include?
   - 1. *(Probe: More Irish examples)*
3. How would you feel about staff in the clinic asking you about the good aspects of your substance use?
   1. *(Probe: How would that make you feel?, comfortable, used to that)*

**Take Home Material**

**If it is ok with you I am now going to move on to ask you about the last step of the treatment that is giving patients take home material...**

- Show client a copy of proposed take home material (feedback report card, substance risk cards)
- Explain how take home material is used.

1. From your experience, do you think we should provide take home material?

*(Probe: Would it be used? Would it be helpful? Why?)*

1. Do you think providing the feedback report card to take home is a good idea?

*(Probe: Why? What would you do with it?)*

1. Do you think there providing the substance risk card to take home is a good idea?

*(Probe: Why? What would you do with it?)*

1. We may provide the take home material in a folder like this, do you think the folder is necessary?
2. Can you think of anything else that would be a good idea to give patients to take home with them?

**Wrap Up**

**We are just at the end now I only have one last question...**

1. Is there anything that has not been mentioned here that you would like to share?

**Thank you again for your time and willingness to participate**
