# Supplementary material for: Tailoring a brief intervention for illicit drug use and alcohol use in Irish methadone maintained opiate dependent patients: a qualitative process
Source: BMC Psychiatry. 2016 Nov 3;16:373. doi: 10.1186/s12888-016-1082-4 (PMC5094097; doi:10.1186/s12888-016-1082-4)
Supplement: Additional file 2: — Screening Led Brief Intervention Training Manual. Screening Led Brief Intervention Reference Manual. Training manual developed as a part of this qualitative process. (DOCX 5115 kb) [file 12888_2016_1082_MOESM2_ESM.docx]

**
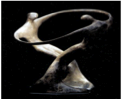
**

**Screening Led Brief Intervention Reference Manual**

**Research Team: Joe Barry, Catherine Darker, Eamon Keenan, Brion Sweeney, Rolande Anderson, and Lucy Whiston**

**
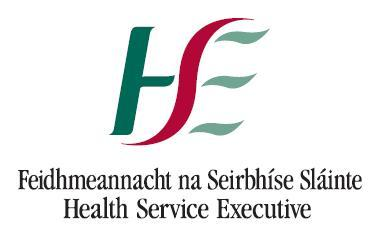

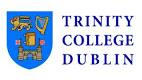

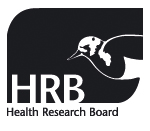
**

**Research Team**

Professor Joe Barry,

Department of Public Health and Primary Care,

Trinity College Dublin,

Dublin,

Ireland.

Assistant Professor Catherine Darker,

Department of Public Health and Primary Care,

Trinity College Dublin,

Dublin,

Ireland.

Dr. Eamon Keenan,

Health Services Executive,

Dublin North Leinster Drug Service,

Dublin,

Ireland.

Dr. Brion Sweeney,

Department of Public Health and Primary Care,

Trinity College Dublin,

Dublin,

Ireland.

Mr. Rolande Anderson,

The Charlemont Cinic,

Dublin 2,

Ireland.

*Ms. Lucy Whiston,

Department of Public Health and Primary Care,

Trinity College Dublin,

Dublin,

Ireland.

01- 8961643

whistonl@tcd.ie

*Main contact

**Contents**

**Glossary of Terms_____________________________________________________________ 5**

**Background __________________________________________________________________ 6-7**

The Screening Tool........................................................................... 6

Brief Intervention.............................................................................. 6

Screening Led Brief Intervention Structure ..................................... 7

**1. Consent ___________________________________________________________________ 8**

**2. Ask_______________________________________________________________________ 9-12**

**3. Advise_____________________________________________________________________ 13**

**4. Assess_____________________________________________________________________ 14**

**5. Assist_____________________________________________________________________ 15**

**6. Arrange___________________________________________________________________ 15**

**7. Motivational Interviewing____________________________________________________ 16-17**

7.1 Motivational Interviewing................................................................. 16

7.2 Motivational Interviewing Skills and Principles............................... 17

**8. Appendices**

Appendix A: Screening Tool Effectiveness......................................... 18

Appendix B: Brief Intervention Effectiveness..................................... 19- 23

Appendix C: Patient Information Sheet............................................... 24

Appendix D: Patient Consent Form..................................................... 25

Appendix E: Screening Tool Response Card....................................... 26

Appendix F: Screening Tool................................................................ 27-33

Questionnaire................................ 27- 31

Risk Score Calculator................... 32

Feedback Report Card.................. 33

Appendix G: Substance Risk Card....................................................... 34-43

Appendix H: Substance Risk Card Photos .......................................... 44-46

Appendix I: Readiness to Change Rulers........................................... 47

Appendix J: Pros and Cons of Alcohol/Drug Use Prompts ............... 48-49

Appendix K: Motivational Interviewing.............................................. 50

Appendix L: Screening Lead Brief Intervention Example.................. 51-56

Appendix M: References...................................................................... 57-58

**Glossary of Terms**

**Screening Tool (ASSIST):** The screening tool is the Alcohol, Smoking and Substance Involvement Screening Test (ASSIST). It is an eight-item screening tool developed by the World Health Organisation, which investigates individuals' lifetime and current substance use and the associated risks and problems for each substance.

**Brief Intervention (BI):** Brief interventions are an evidence-based group of psychosocial strategies

which aim to reduce substance use and/or harms related to substance use, in a time efficient manner. A BI is underpinned by the Stages of Change, Motivational Interviewing and the FRAMES technique.

**Screening led BI:** This is a short intervention lasting 3-15 minutes given to patients who have been administered the ASSIST by a clinician.

**Motivational Interviewing:** MI is a collaborative patient centred, goal orientated approach for facilitating change by exploring and resolving ambivalence about the patient's substance use. This is achieved using techniques such as developing discrepancies, rolling with resistance, expressing empathy, and supporting self-efficacy.

**F.R.A.M.E.S.:** Refers to the use of:

**F**eedback based on the results of the screening test, **R**esponsibility for change lies with the individual,

**A**dvice giving,

**M**enu of change options,

**E**mpathic counselling style,

**S**elf-efficacy of the patient.

**Background**

A screening led BI is an intervention developed by the World Health Organisation (WHO) to change patients' problematic substance use. This is done in two main stages the screening tool, the Alcohol, Smoking and Substance Involvement Screening Test (ASSIST), followed by a BI.

**The Screening Tool (The ASSIST)**The screening tool is the Alcohol, Smoking and Substance Involvement Screening Test (ASSIST) 3.1. The ASSIST is an eight-item screening tool, which investigates the individual’s lifetime and current substance use and the associated problem for each substance ^1^. Questions are asked in relation to frequency of use, cravings, the impact of substance use, ability to meet expectations, concern of friends, relatives and health care workers, attempts to cut down on substance use and risky behaviour such as intravenous use. Developed under the auspices of the World Health Organisation by an international group of addiction researchers and clinicians, it was designed to be used across a range of countries and cultures in a primary care setting as a screening tool in conjunction with a BI ^2^. The ASSIST provides a risk score for each substance and for substance use overall. Scores are grouped into low, moderate and high to determine the level of intervention: treatment as usual, BI or BI and referral to specialist treatment. Since its development, considerable testing has demonstrated the ASSIST to be a valid and reliable instrument suitable to be used in a variety of countries and settings. Further evidence for the effectiveness of the screening tool can be found in Appendix A: Screening Tool Effectiveness (p. 18).

**Brief Interventions**
‘Brief interventions can be defined as a group of strategies which aim at reduction of substance use and/or harm related to substance, in cost-effective and time–efficient manner, by imparting brief or minimal advice/counselling to the users of alcohol, tobacco or other drugs’ ^3^. Developed by the WHO an ASSIST led BI is generally short in duration and can be conducted by non-addiction specialists. The aim is to help the patient to identify substance use problems, understand the risks involved and motivate those at risk to reduce or abstain from their substance use ^4^. BIs consist of a group of evidence based psychosocial strategies underpinned by a theoretical framework drawing primarily on principles of Prochaska and DiClementes' ^5^ Stages of Change, and Miller and Rollnicks' ^6^ motivational interviewing incorporating the FRAMES technique. Evidence for the effectiveness of BIs is outlined in Appendix B: The Effectiveness of Brief Intervention (p. 19).

**Screening Led Brief Intervention Structure**

**1.** Consent Explain consent and ensure patient completes the consent *30 sec - 1 min*

(Research action) form for data to be given to the research team in Trinity.

**⇓**

**2. A**sk Screen patient using the screening tool. *6-8 mins*

**⇓**

**3. A**dvise Present screening results. *1-2 mins*

Emphasise individual responsibility for change.

Advise reduction or abstinence where appropriate.

**⇓**

**4. A**ssess Readiness to change - Patients' level of concern. *1-2 mins*

- Help weigh up pros and cons

**⇓**

**5. A**ssist Summarise and reflect back. *1-2 mins*

Negotiate reasonable goals.

**⇓**

**6. A**rrange Refer and arrange further help and treatment if necessary. *30 sec - 1 min*

Provide take home material.

Remind about three month follow up.

**⇓**

**7.** Return Return completed consent form and screening tool to designated box.

(Research action)

* *Please note suggested times are only a rough guideline.*

**1. Consent**

**(Research Action)**

* Consent for data to be given to the research team *

- Take the patient through the information sheet in Appendix C: Patient Information Sheet (p. 24). Explain the nature of the project; what the research questions are, the purpose of the study and why it is important.
- Ensure patients understand participation is voluntary and does not impact on their treatment.
- Explain that the questionnaire will be repeated 3 months later.
- Take the patient through the consent form in Appendix D: Patient Consent Form (p. 25). Paraphrase where necessary while asking if the patient has any questions or issues they would like to raise.
- Emphasise confidentiality to the research and treatment teams.
- Ask the patient to sign the consent form, which will be countersigned by you the clinician.

**2. Ask**

Once consent has been obtained the next step is to ask the patient about their substance use through the screening tool. The process of administering the screening tool is outlined in Figure 1 below.

***Figure 1: Administering the Screening Tool***

| Phase One Introduce the Screening Tool |
| --- |

⇓

| Phase Two Screening Tool |
| --- |

↓

| Substances ever used (Q1) |
| --- |

↓

| Substance used in the last 3 months (Q2)  *Only ask about substances used in Q1* |
| --- |

**If no to all skip to Q6** →

**If yes to any**

**↓**

| Current Use (Q3, Q4 + Q5) |
| --- |

↓

| Lifetime substance use (Q6 +Q7)  *Ask about all substances used in Q1* |
| --- |

↓

| Intravenous use (Q8) |
| --- |

⇓

| Phase Three Calculate Risk Score |
| --- |

**Phase One: Introduce the screening tool**

- Give the patient the response cards (Appendix E: Screening Tool Response Card, p. 26).
- Explain the list of substances and ensure that they are referred to by names that are familiar to the patient.
- Explain that the questions cover lifetime use and use in the last three months.
- Explain that the questions relate to non-prescription use only (i.e. street methadone, street benzos)
- Explain confidentiality.

***Sample Introduction***

''The following questions ask about your experience of using alcohol and other drugs across your lifetime and in the past three months. These substances can be smoked, swallowed, snorted, inhaled or injected (show response card).”

“For this interview, we will only record substances which *have not been prescribed by a doctor*. For example, we will only record street benzos or street methadone not benzos prescribed by your doctor or the methadone you receive here at the clinic''

**Phase Two: Administer the Screening Tool**

Administer the screening tool provided in Appendix F (p. 27) using the good practice tips below.

***Good practice***

- Circle a response for every drug for every question.
- Remind patients of what the categories mean as you progress through the questionnaire.
- Use the same street name for substances as patients use.
- Provide examples and record 'other' drugs where appropriate.
- You may need to rephrase or use prompts for some questions providing examples.
- If patients' answers are inconsistent you need to probe to ensure that the question has been understood.
- Be clear about interpreting and scoring the screening tool before you start. Use the Frequency Response Card in Appendix E (p. 26) as an aid.

***Question Specific Tips***

- Question One (Lifetime use):

**'In your life which of the following have you ever used?'**

*- If a patient reports never using a substance in Question One do not ask about it again.*

- Question Two (Current Use):

**'In the past three months, how often have you used the substances you mentioned?'**

- When explaining the time period of three months it may be helpful to pick an event that may stand out to help the patient e.g. Easter, St. Patricks Day, the May Bank Holiday.

- If a patient reports not using a substance in Question Two do not ask about it again in Questions three, four or five.

- Question Three (Craving):

**'In the past three months, how often have you had a strong desire or urge to use...?'**

- Reflects high risk or dependence with intense craving not mild desire.

- Only record desire for substance used in the last 3 months.

- A high score here is generally consistent with more frequent use, previous problems with the substance and injecting drugs.

- Question Four (Psychosocial impact):

**'During the past three months, how often has your use of 'X' led to health, social, legal or financial problems?'**

- Aims to determine the level of frequency with which substances used in the last 3 months create problems for patients.

- Note that social and occupational problems are included and that health encompasses both physical and mental health.

- You may have to help patients in linking their substance use to associated problems so it is important to prompt and provide examples.

- Question Five (Failed expectations):

**'During the past three months, how often have you failed to do what was normally expected of you because of your use of...?'**

*- Do not ask about tobacco for this question.*

- Investigates if patients are experiencing problems fulfilling their obligations because of the substances used in the last 3 months.

- You may need to prompt and provide examples here such as failing to fulfil obligations due to being intoxicated, recovering from using a substance, waiting for a dealer. - Obligations missed may include missing or performing poorly at work or in education, missing out on pay, failing to maintain family or relationship commitments.

- Question Six (Concern):

**' Has a friend or relative or anyone else ever expressed concern about your use of ...?'**

- Designed to find out if another person (e.g. family, friend, partner, spouse, parent, child, doctor, employer, etc.) has expressed concern about the patient's substance use.

- Ask about all substances nominated in Q1.

- Question Seven (Cut down/control):

**' Have you ever tried and failed to control, cut down or stop using...?'**

- An indicator of dependence or high-risk use and is designed to identify loss of control over substance use.

- Ask about all substances nominated in Q1.

- Successful attempts to cut down are not recorded.

- If the patient has made several attempts to cut down and was eventually successful the clinician needs to record the last unsuccessful attempt.

- Question Eight (Intravenous use):

**' Have you ever used any drug by injection?'**

- If the patient has injected drugs in the last 3 months you need to ascertain the frequency and pattern of intravenous use.

**Phase Three: Calculate Risk Score**

The Risk Score is calculated by adding all questions from **two to seven** together. This score provides a measure of use and problems over the previous three months for each of the substances covered by the screening tool. The Risk Score Calculator in Appendix F (p. 32) can be used to help you with this. The score for each substance is then recorded in the Feedback Report Card (Appendix F, p. 33). The screening tool risk score determines the most appropriate intervention for that level of use as outlined in Figure 2 below. *No Risk Score will be calculated for tobacco.*

***Figure 2: Linking Screening Tool Risk Scores With Action***

| Substance | Action | | |
| --- | --- | --- | --- |
|  | **Feedback** | **Feedback and Brief Intervention** | **Brief Intervention and referral** |
| Alcohol Risk Score | 0-10 | 11-26 | 27+ |
| All Other Substances Risk Score | 0-3 | 4-26 | 27+ |
| Intravenous use |  | Less than 4 times in the past 3 months | More than 4 times in the past three months |

**3. Advise**

**Provide Feedback**

- Identify target substance.
- If two or more substances score an equally high Risk Score negotiate with the patient which substance will be targeted.

**Emphasise Individual Responsibility**

- Patients are responsible for their own decisions and this needs to be reiterated to patients during a BI as maintaining personal control is an important motivating factor in achieving change.

**Advise Reduction or Abstinence Where Appropriate**

- Advise a reduction of substance use or abstinence where appropriate.
- Ensure you employ a Motivational Interviewing style, not engendering resistance (p. 16).
- Communicate the risks associated with the highest scoring substance using the relevant Substance Risk Card in Appendix G (p. 34) and if necessary the intravenous use card. For those with literacy problems use the pictures on the back of the Substance Risk Cards depicted in Appendix H: Substance Risk Card Photos (p. 44).

**4. Assess**

**Assess Patient's Level of Concern**

Assess readiness to change using the readiness and confidence rulers on the readiness to change sheet in Appendix I (p. 47). The 'Readiness Ruler' is a simple way to find out how important the patient thinks it is to reduce their substance use and to encourage patients to talk about reasons for and against change. Patients are asked on a scale of one to ten, where one is not at all important and ten is extremely important, how important it is for them to stop/cut down their drinking/drug use. The 'Confidence Ruler' is a similar scale, which assesses how confident a patient is that they can cut down or stop their substance use. Patients are asked on a scale of one to ten, where one is not at all confident and ten is extremely confident, how confident they are that they can stop/cut down on their drinking/ drug use ^6^.

It is not necessary to actually show the patient the ruler, but it may be helpful, especially for patients with low literacy and numeracy. For some patients it may be enough to just describe the scale using words like those provided on the rulers e.g. not at all important, extremely confident.

**Help Patients Weigh up Pros and Cons**

Use patients' answers for the readiness and confidence rulers and score on the screening tool to foster talk about the reasons for and against their substance use. Encourage them further with questions such as:

- 'What would make a 3 become a 4?'
- 'Why did you choose 5 and not 4?'
- 'How do you feel about the score you received?'
- 'Why do you want to cut down/stop your use of 'X'?'
- 'What reasons are against you cutting down/stopping your use of 'X'?'
- 'How does your use of 'X' impact on your life?'
- 'How does your use of 'X' help you?
- 'What have you missed out on because of your use of X?'

Examples are provided in Appendix J: Pros and Cons for Drug/Alcohol Use Prompts (p. 48) to elicit reasons for and against substance use change from patients. These examples are only provided for prompting, the focus should be on the reasons put forward by the patient.

**5. Assist**

**Summarise and reflect:**

- Summarise and reflect back to the patient what they have just said about the good and less good things about their substance use.
- This is a simple, effective way of acknowledging the experience of the patient and preparing them to move on.
- If patients feel listened to they are more likely to change.

**Negotiate goals:**

- Help the patient to set a realistic and achievable goal to work towards in changing their substance use.
- Check to make sure that this goal is realistic for them.
- Ask the patient to suggest ways in which they can go about achieving this goal.

**6. Arrange**

**Refer and arrange if necessary:**

- Patients with a score above 27 for their target substance, or who have injected more than four times in the past three months, need to receive a referral for further in-house treatment and support.

**Provide take home material:**

- Give the patient a take home folder with a copy of their feedback report card and relevant substance risk card and if necessary intravenous use card to take away with them.
- The written information can strengthen and consolidate the effects of the BI. The feedback report card serves as a reminder of the patient's score. The substance risk card reminds the patient of the risks associated with their target substance that has been the focus of the BI.

**Remind about three month follow up**

- Remind patients that we will return to check in with them in three months time.

**7. Motivational Interviewing Style**

**7.1 Motivational Interviewing**

A key tool in administering a BI is the use of a Motivational Interviewing (MI) style. Motivation is 'a state of readiness to change, which fluctuates and can be influenced by others ^7^. MI is underpinned by the hypothesis that a change in motivation needs to come from within an individual and the way in which patients are spoken to can either enhance or minimise motivation ^7^. As such, this is a patient centred and directive approach ^8^. The aim is to help patients to work through ambivalence about behaviour change and so progress from one stage of change to the next, see Figure 3 below. Progression is achieved through collaborative interactions between the clinician and patient with the service user articulating for themselves the reasons for concern and arguments for change ^9, 10^. Further information on motivational interviewing skills and principles is provided in Appendix K: Motivational Interviewing (p. 50).

***Figure 3. Prochaska and DiClementes' Wheel of Change (Original and Plain English)***

Adapted from ^11.^

**7.2 Motivational Interviewing Skills and Principles**

**Spirit:** Patient centred and directed approach, which encourages the process of change by exploring ambivalence and facilitating patients to implement change.

**Principles: *Express Empathy-*** Using a non-judgemental approach while attempting to understand the patient's point of view, avoiding confrontation, and listening reflectively.

***Develop discrepancies-*** Elicit and amplify discrepancies between patient's words, actions and goals.

***Roll with resistance-*** Understand that ambivalence and resistance are normal parts of the process of change. When resistance emerges clinicians should reframe or reflect it rather than opposing it.

***Collaborative-*** The conversation should run between two equals with the aim of evoking the reasons for change from the patient.

***Support self-efficacy-*** Using negotiation and confidence building to increase patients' own belief that something can be done and that changing behaviour is more than possible.

**Skills: *O.A.R.S.*** and elicit change talk.

**O**pen-ended questions • Using open-ended questions that require longer answers and facilitate more conversation.

**A**ffirmations • Include statements of appreciation and understanding to build rapport and support your patient's confidence.

**R**eflective listening • Reflect on the meanings and feelings of the patient using words already used.

**S**ummarise • Summarise to highlight ambivalence and discrepancies and encourage the patient to progress.

***Eliciting change talk*** • Help patients resolve ambivalence by presenting the arguments for change through recognising the disadvantages of staying the same and advantages of change, expressing optimism about change and intentions to change.

**8. Appendices**

**Appendix A: Screening Tool Effectiveness**

The suitability of the ASSIST to be employed internationally has been illustrated in a number of studies. For example, through research in Australia, Brazil, India and the United States, with a sample recruited from primary health care settings, the ASSIST was demonstrated as suitable to be employed across different countries and backgrounds within a broad range of cultural, political and economic systems in which substance-related problems occur ^12^. In addition to this a sample recruited from primary health care and also substance abuse treatment services in nine countries reported that 98% of service users found the ASSIST questions to be non-offensive and 100% of interviewers felt respondents were not offended by questions ^13^.

The reliability and feasibility of ASSIST questionnaire items were assessed in a test-retest trial in nine countries with a general population and an in-treatment sample ^13^. The ASSIST was administered and rated by interviewers and interviewees in terms of comprehension, cultural appropriateness, and ability to elicit an honest answer. Internal consistency reliability was high for most items except tobacco. Average Kappas ranged from a high of 0.90 to a low of 0.58. This is in line with feedback through interviews in which 78% of service users indicated that none of the questions were difficult to understand, 77% of interviewers reported that the ASSIST was easy to administer and 97% of interviewers reported feeling that service users were interested in the ASSIST ^13.^

The validity of the ASSIST was tested in a variety of treatment settings and countries illustrating that 'the ASSIST shows excellent concurrent, construct, predictive and discriminative validity and can adequately screen for low, moderate and high risk substance use for any substance' ^1^. Furthermore, the ASSIST was shown to be valid on all counts when examined against other gold standards such as the AUDIT, MAP and the RTQ-Smoking score ^1^.

**Appendix B: Brief Intervention Effectiveness**

The present literature on BIs examines the impact of this treatment option in a number of areas.

***Reduction in Substance Use***It is clear that BIs typically result in a reduction in substance use. The effectiveness of a BI linked with the ASSIST screening tool on illicit drug use was examined with a sample of over seven hundred service users recruited from primary care settings in Australia, Brazil, India and the United States. After receiving an intervention, 82.8% of the sample attempted to reduce their illicit drug use with 60.2% succeeding in maintaining this reduction for an average of 11.2 weeks at the three month follow up ^14^. This positive relationship between BIs and substance use reduction has also been demonstrated by numerous other sources. For example, meta-analysis of twenty-two randomised control trials (RCTs) depicted an average reduction of the equivalent of four to five units of alcohol a week after a BI was administered at one year follow up ^15^. The association between BIs and reduced alcohol consumption has been depicted in numerous studies ^15-20^. The positive relationship between BIs and illicit substance use is also clearly evident within presently available sources. To date the impact of BIs on a variety of illicit substances has been examined and shown to be effective in relation to amphetamines ^21^ benzodiazepines ^22^, cannabis ^23-25^, cocaine ^26, 27,^ opiates ^28^ and illicit substances in general ^12, 14, 29.^

***Abstinence***
While it is clear from the literatures that BIs typically result in a reduction in substance consumption, there does not appear to be a consensus in relation to abstinence. An RCT was conducted to test the impact of a single structured encounter targeting cessation of cocaine and heroin use. At follow up, the intervention group was more likely to be abstinent from heroin and cocaine (17.4% vs. 12.8%) ^26^. Australian research analysed BIs with 229 cannabis users and found that the treatment groups reported a greater proportion of days abstinent than the delayed treatment group ^24^.

Nonetheless, the percentage of those who achieve abstinence is predominantly low and often not significant with the typical outcome from a BI being substance use reduction not cessation. This has been illustrated in an Australian based study, which explored whether BIs are feasible among regular amphetamine users recruited from the general population, but also treatment settings, and assessed the effectiveness of interventions. BIs were shown to be effective in reducing amphetamine use and the intervention group were significantly more likely to be abstinent at six month follow up. However, the typical participant was still using amphetamines at least weekly, cannabis three times a day, nineteen cigarettes and three and a half types of drugs a month ^21^. This conclusion was corroborated by a study with young people in London. This study found that while those randomised to the treatment group reduced their substance use this was mainly through moderation of use rather than cessation of use ^30^.

***Addiction Severity***
A number of studies have examined the impact of BIs on service users' dependence and addiction severity. A BI is demonstrated as leading to a reduction in service users' dependence, associated problems and addiction severity stemming from a reduction in substance consumption. An Australian based study focusing on the use of BIs with cannabis users recruited 229 participants from the general population. Participants were randomly assigned to delayed treatment, one intensive BI or six sessions of motivational interviewing and relapse prevention. The treatment groups demonstrated a significantly lower Severity of Dependence score, as assessed by the Addiction Severity Index, and were less likely to be concerned about their control over cannabis and any related problems ^24^. Similar trends have also been reported for alcohol ^31^, marijuana ^23^ and ecstasy ^32^. In another Australian based study with ecstasy a significant decrease in the number of dependence symptoms was demonstrated at three month follow up ^32^.

***Level of Motivation and Self-Efficacy***
There has been a limited focus on research pertaining to the impact of BIs on service users' level of motivation and self-efficacy. The impact of a brief motivational intervention on readiness to change and self-efficacy was investigated with 122 opiate users attending a methadone programme ^28^. Service users were randomly allocated to either brief motivational therapy or an educational pack with follow up at three and six months. This study illustrated that those who received a BI were more likely to make progression in relation to the stages of change, set goals representing greater commitment, and reported more positive outcome expectancy with higher levels of self-efficacy. At the three-month follow up, 37% of those who received a BI were classified as actioneers while the majority of the control group (47%) remained pre-contemplators. However, impact on service users' stage of change and level of self-efficacy was not sustained at six-month follow up. Similar results were also reported in a study of alcohol use following a brief intervention or simple advice. Ninety men in North India, defined by the AUDIT as having problematic drinking, were recruited from a previous community based house-to-house survey study and reported a definite change from pre-contemplation to contemplation. At baseline, of those assigned to the BI group 37.8% were pre-contemplators, 26.6% contemplators, and 15.5% actioneers. By one month follow up there were no participants in pre-contemplation, 31.2% in contemplation and 62.7% in action in the BI group. However this pattern was not sustained at follow up three months after the baseline at which 54.7% of those in the treatment group were in pre-contemplation, 30.9% in contemplation and 16.6% in action ^31^.

***Health***

Focusing on the individual, for the most part, the literature presents a positive impact of BIs on service users' health. For example, two thirds of service users who received a BI with screening through the ASSIST as part of the WHO development of BIs reported a positive influence on their health behaviour ^1^. Further detail is provided on this outcome in subsequent research in which 82.5% of service users who received an ASSIST linked BI, reported a positive influence on their health behaviour. More specifically, improvements included better health with more energy, and improved appetite, sleep and drug use or drug use attitude ^14^. Similar findings have also been presented by numerous other sources ^20, 25, 31^.

A prominent finding in this area is that there is no psychological distress or harm caused by a BI. An RCT of two BIs was conducted with a population of long-term benzodiazepine users recruited from a GP setting. No evidence was reported that BIs increased psychological distress or had adverse effects on general health ^33^. This confirms a previous finding from a study with a vulnerable population with high rates of past depression, anxiety and attempted suicide who demonstrated no psychological distress or harm stemming from a BI ^22^.

On a wider level, the impact of BIs on healthcare systems has undergone considerable examination, the public health impact of which has been described as 'potentially enormous' ^34^. Research to date has proposed a positive impact on healthcare systems through a reduction in the burden of illnesses associated with substance use and addiction ^12, 14^. Furthermore, BIs have been proposed as a possible option in developing and improving current healthcare systems. In an international study of the ASSIST, emphasis was placed on the need to adapt the current traditional specialised care model for substance abuse treatment to a more integrated approach with an expansion of services to less severe cases and populations of risk ^14^. BIs were proposed as a possible option to employ in this transition, particularly through opportunistic screening ^14^. Finally, available research has also alluded to an impact on healthcare systems through an influence of BIs on subsequent substance abuse treatment ^17^.

***Social Functioning***

The impact of BIs on service users' social functioning is a secondary outcome arising in a limited number of sources within the current literature. An Australian based study with self-defined cannabis users with a pre and post-test design reported improved social functioning stemming from substance use reduction as a result of a BI. Improvements were seen in relation to relationships with family and friends, interest in sports, hobbies and other activities, employment and job performance and were maintained at the one and three month follow up ^25^. However, beyond this it would appear that reductions in substance use do not necessarily go hand in hand with improvements in secondary outcomes such as social functioning ^16, 19^.

***Subsequent Treatment***
It has been argued that BIs provide preparation and motivation for future treatment ^17^. The impact of BIs on service users' subsequent treatment experiences such as treatment entry, engagement and length of stay has been explored in a number of sources and generally as a secondary outcome. A brief motivation intervention was developed for utilisation within a medical setting and reported increased entry and engagement in subsequent more intensive treatment ^9^. There are numerous other sources which put forward that BIs are most effective when used as initial treatment or as an enhancement to more intensive substance abuse treatment ^8, 35^. Beyond treatment entry, a systematic review of the literature on BIs surmised that ‘Brief interventions can impact treatment participation, and through this or other mechanisms exert a beneficial impact on post-treatment outcomes’ ^17^. This has been illustrated through research with cocaine dependent patients in a detoxification and relapse prevention programme ^27^. From this research, it was evident that patients who completed a brief motivational interview were more likely to be abstinent from cocaine at the start of relapse prevention treatment, demonstrated increased use of behavioural coping strategies and were less likely to use cocaine throughout treatment.

***Comparisons with alternative treatments***
In presently available research BIs have been compared to a number of other treatment alternatives. The focus has mainly been on BIs in comparison to 'treatment as usual' (TAU), less intensive treatment or more intensive treatment. In general BIs compare favourably. ‘It appears that brief interventions for alcohol problems: (1) are usually significantly more effective that no intervention; (2) commonly show similar impact to that of more extensive interventions...’ ^17^.

The primary focus is on BIs as opposed to TAU with the majority of studies to date including a control group, who receive TAU or a delayed intervention after the study is complete, and an intervention group. A review of BIs in substance abuse illustrated that when compared with TAU BIs were found to be significantly more effective ^3^. This is supported in meta-analysis ^17^ and international research ^14^. Furthermore, in a sample of problem alcohol using males when BIs were compared with less extensive simple advice significant differences were noted. Better three-month outcomes were reported for service users who received a BI with a greater decrease in severity of dependence and improvements in physical and psychological quality of life ^31^.

In comparisons between BI and more intensive treatments, results are also favourable. A meta-analysis of studies comparing BIs with either a control or extended treatment found little difference between BIs and extended treatment ^35^. This is in line with a previous systematic review which found BIs to be as effective as more intensive psychosocial treatments ^17^.

**Appendix C: Patient Information Sheet**

**Patient Information Sheet**

**Screening and Brief Interventions for Illicit Drug Use and Alcohol Use in Methadone Maintained Opiate Dependent Patients: A cluster randomised controlled trial feasibility study.**

**Research Team:** Prof Joe Barry, Assistant Prof Catherine Darker, Dr Eamon Keenan, Dr Brion Sweeney, Ms. Lucy Whiston

**What is the aim of this research?** We are doing a survey of drink and drug use in methadone patients. We want to check if a Brief Intervention treatment works for patients who have a problem with drink and drugs. The Brief Intervention will be a like a chat with a member of staff in the clinic who will try to help you to think about your drug and alcohol use and maybe find ways of changing your use. We will ask you questions about your drink and drug use now and we will ask you the same questions again in three months time.

**What steps will be taken to make sure that any information you give us is confidential?** If you agree to take part in the study your information will be given to the research team from Trinity College Dublin. We will take your name off your information, which may include your urine results, we will then give your information an anonymous i.d code (such as P102). Your information will be used to check if a Brief Intervention works.

**What are the possible risks of taking part in this study?** There are no risks to taking part in this study.

**What are the possible benefits of taking part in this study?** Taking part might help you to look at your drink and drug use and might help other patients in the future.

**Do I have to take part?** No, you do not have to take part in this study and even if you agree to be in the study now, you can change your mind later. If you decide to take part you will need to sign a consent form. If you do not want to take part or you leave the study this will not affect your treatment in any way.

**What care is available if I do not want to take part in the study?** You do not have to be part of the study to get treatment for your drink and drug problems.

**What will happen to the study results?** The study results may help doctors and researchers to make better decisions about treating patients. The results of the study may be used for publication in a medical journal. Your information will not be linked back to you in any way.

**More detail:** If you have any questions call Lucy Whiston on 01-8961643.

**Appendix D: Patient Consent Form**

**Consent Form**

**Agreement for information to be passed onto research team:**

**Screening and Brief Interventions for Illicit Drug Use and Alcohol Use in Methadone Maintained Opiate Dependent Patients: A cluster randomised controlled trial feasibility study.**

The study has been explained to me. I have had a chance to ask questions about the study and what it involves. I know that I do not have to take part. I know that I am free to leave the study at any time. I know that my decision not to take part or to leave the study will not have an effect on treatment for my drink or drug use. I agree to be surveyed again in three months time. I know that researchers might access my treatment records including my urine analysis results. I am happy for details about my drink and drug use to be given to the research team. I am happy that details of my taking part in this study and my information will be kept strictly confidential to the research and treatment teams.

I, the undersigned, hereby consent to take part in the study as outline in the information sheet.

_________________________________ (Please sign here)

_________________________________ (Please print your name here)

Date: _______________________ Time: __________________

***Statement of clinician's responsibility:*** I believe the participant understands my explanation and has freely given permission for their information to be passed onto the research team.

Clinician's Name:

Clinician's Signature:

Date:

**Appendix E: Screening Tool Response Card**

| **Response Card: Substance** |
| --- |
| a. Tobacco products (cigarettes, smokes, chewing tobacco, cigars, etc.) |
| b. Alcoholic beverages (beer, wine, spirits, cider, cans, etc.) |
| c. Cannabis (hash, weed, skunk, blow, shit, dope, marijuana, pot, grass, etc.) |
| d. Cocaine (coke, crack, charlie, snow, etc.) |
| e. Amphetamine-type stimulants (ecstasy, E, disco, mitsubishi, wizz, uppers, yokes, shamrocks, speed, diet pills, bombs, etc) |
| f. Inhalants (gas, nitrous, glue, petrol, paint thinner, etc.) |
| g. Sedatives or sleeping pills (street benzos, diazepam (bluies, yellows, D5s, D10s), valium, rophypnol (ro ros, roach), kava, GHB, liquid ecstasy, zimmos, dalmane (dolly), etc.) |
| h. Hallucinogenics (LSD, acid, mushrooms, PCP, special k (angel dust, vitamin K, kit kat), ketamine, etc.) |
| i. Opioids (heroin (gear, smack, junk), street methadone (molly, phy, high phy, magpie), morphine, codeine, solpadeine, GGs, DF118 (DFs), etc.) |
| j. 'Headshop products' (snowblo, party pills, go-E, energy, silver bullets, snowstorm, saliva, spice, spice gold, spice diamond, mephedrone, hurricane charlie, snow, vanilla sky, meow meow, meph, doves, summer daze, loved up, cherries, XXX, BZP, trance, beanz, vegas nights, purple ohms, shrooms, trip E happy caps, kratom, xscape, krypton etc.) |
| k. Other- specify (steroids, etc.) |

| **Response Card: Frequency** |  |
| --- | --- |
| Last 3 months (Questions 2 to 5)  • Never: not used in the last 3 months.  • Once or twice: 1 to 2 times in the last 3 months.  • Monthly: roughly 1 to 3 times per month over the last 3 months.  • Weekly: 1 to 4 times per week in the last 3 months.  • Daily or almost daily: 5 to 7 days per week in the last 3 months. | Lifetime (Questions 6 to 8)  • No, never.  • Yes, but not in the last 3 months.  • Yes, in the last 3 months. |

**Appendix F: ASSIST Screening Tool**

| For office use only: ID number _________________ |
| --- |

**ASSIST / Screening Tool**

**Date: __________________________ Clinician Name: __________________________**

**Patient Name: __________________________ Patient DOB: ___________________________**

**Action Taken:**  No action  Brief Intervention  Brief Intervention and referral

**Target Substance:**  Alcohol

Cannabis

Cocaine

Amphetamine- type stimulants

Inhalants

Sedatives

Hallucinogenics

Opioids

'Headshop products'

Other - please specify _______________

**Introduction:**

- Questions about alcohol and other drug use.
- Across your lifetime and in the last three months.
- Substances can be smoked, snorted, injected or taken in the form of a pill (show response card).
- Prescribed medication will not be recorded.
- Answers are confidential to the treatment and research teams.
- Honest answers are important, as we need to know what you actually do.

Question 1

| In your life, which of the following substance have you ever used? (NON MEDICAL USE ONLY) |  |  |
| --- | --- | --- |
| a. Tobacco products (cigarettes, smokes, chewing tobacco, cigars, etc.) | No | Yes |
| b. Alcoholic beverages (beer, wine, spirits, etc.) | No | Yes |
| c. Cannabis (hash, skunk, marijuana, pot, grass, etc.) | No | Yes |
| d. Cocaine (coke, crack, charlie, snow etc.) | No | Yes |
| e. Amphetamine type stimulants (E, ecstasy, speed, diet pills, etc.) | No | Yes |
| f. Inhalants (gas, nitrous, glue, petrol, paint thinner, etc.) | No | Yes |
| g. Sedative or sleeping pills (benzodiazepine, rohypnol, zimmos etc.) | No | Yes |
| h. Hallucinogens (LSD, acid, mushrooms, PCP, special k, etc.) | No | Yes |
| i. Opioids (heroin, morphine, methadone, codeine, etc.) | No | Yes |
| j. Headshop products (snowblo, spice, meow meow, BZP, etc.) | No | Yes |
| k. Other- specify | No | Yes |

Probe if all answers are negative: 'Not even when you were in school?

***If 'No' to all items, stop interview.***

***If 'Yes' to any of these items, ask Question 2 for each substance ever used.***

Question 2

| In the past three months, how often have you used the substances you mentioned (FIRST DRUG, SECOND DRUG, ETC)? | Never | Once or twice | Monthly | Weekly | Daily or almost daily |
| --- | --- | --- | --- | --- | --- |
| a. Tobacco products (cigarettes, smokes, chewing tobacco, cigars, etc.) | 0 | 2 | 3 | 4 | 6 |
| b. Alcoholic beverages (beer, wine, spirits, etc.) | 0 | 2 | 3 | 4 | 6 |
| c. Cannabis (hash, skunk, marijuana, pot, grass, etc.) | 0 | 2 | 3 | 4 | 6 |
| d. Cocaine (coke, crack, charlie, snow etc.) | 0 | 2 | 3 | 4 | 6 |
| e. Amphetamine type stimulants (E, ecstasy, speed, diet pills, etc.) | 0 | 2 | 3 | 4 | 6 |
| f. Inhalants (gas, nitrous, glue, petrol, paint thinner, etc.) | 0 | 2 | 3 | 4 | 6 |
| g. Sedative or sleeping pills (benzodiazepine, rohypnol, zimmos etc.) | 0 | 2 | 3 | 4 | 6 |
| h. Hallucinogens (LSD, acid, mushrooms, PCP, special k, etc.) | 0 | 2 | 3 | 4 | 6 |
| i. Opioids (heroin, morphine, methadone, codeine, etc.) | 0 | 2 | 3 | 4 | 6 |
| j. Headshop products (snowblo, spice, meow meow, BZP, etc.) | 0 | 2 | 3 | 4 | 6 |
| k. Other- specify | 0 | 2 | 3 | 4 | 6 |

***If 'Never' to all items in Question 2, skip to Question 6.***

***If any substance in Question 2 was used in the previous three months, continue with Questions 3, 4, and 5 for each substance used.***

Question 3

| In the past three months, how often have you had a strong desire or urge to use (FIRST DRUG, SECOND DRUG, ETC.)? | Never | Once or twice | Monthly | Weekly | Daily or almost daily |
| --- | --- | --- | --- | --- | --- |
| a. Tobacco products (cigarettes, smokes, chewing tobacco, cigars, etc.) | 0 | 3 | 4 | 5 | 6 |
| b. Alcoholic beverages (beer, wine, spirits, etc.) | 0 | 3 | 4 | 5 | 6 |
| c. Cannabis (hash, skunk, marijuana, pot, grass, etc.) | 0 | 3 | 4 | 5 | 6 |
| d. Cocaine (coke, crack, charlie, snow etc.) | 0 | 3 | 4 | 5 | 6 |
| e. Amphetamine type stimulants (E, ecstasy, speed, diet pills, etc.) | 0 | 3 | 4 | 5 | 6 |
| f. Inhalants (gas, nitrous, glue, petrol, paint thinner, etc.) | 0 | 3 | 4 | 5 | 6 |
| g. Sedative or sleeping pills (benzodiazepine, rohypnol, zimmos etc.) | 0 | 3 | 4 | 5 | 6 |
| h. Hallucinogens (LSD, acid, mushrooms, PCP, special k, etc.) | 0 | 3 | 4 | 5 | 6 |
| i. Opioids (heroin, morphine, methadone, codeine, etc.) | 0 | 3 | 4 | 5 | 6 |
| j. Headshop products (snowblo, spice, meow meow, BZP, etc.) | 0 | 3 | 4 | 5 | 6 |
| k. Other- specify | 0 | 3 | 4 | 5 | 6 |

Question 4

| During the past three months, how often has your use of (FIRST DRUG, SECOND DRUG, ETC.) led to health, social, legal or financial problems? | Never | Once or twice | Monthly | Weekly | Daily or almost daily |
| --- | --- | --- | --- | --- | --- |
| a. Tobacco products (cigarettes, smokes, chewing tobacco, cigars, etc.) | 0 | 4 | 5 | 6 | 7 |
| b. Alcoholic beverages (beer, wine, spirits, etc.) | 0 | 4 | 5 | 6 | 7 |
| c. Cannabis (hash, skunk, marijuana, pot, grass, etc.) | 0 | 4 | 5 | 6 | 7 |
| d. Cocaine (coke, crack, charlie, snow etc.) | 0 | 4 | 5 | 6 | 7 |
| e. Amphetamine type stimulants (E, ecstasy, speed, diet pills, etc.) | 0 | 4 | 5 | 6 | 7 |
| f. Inhalants (gas, nitrous, glue, petrol, paint thinner, etc.) | 0 | 4 | 5 | 6 | 7 |
| g. Sedative or sleeping pills (benzodiazepine, rohypnol, zimmos etc.) | 0 | 4 | 5 | 6 | 7 |
| h. Hallucinogens (LSD, acid, mushrooms, PCP, special k, etc.) | 0 | 4 | 5 | 6 | 7 |
| i. Opioids (heroin, morphine, methadone, codeine, etc.) | 0 | 4 | 5 | 6 | 7 |
| j. Headshop products (snowblo, spice, meow meow, BZP, etc.) | 0 | 4 | 5 | 6 | 7 |
| k. Other- specify | 0 | 4 | 5 | 6 | 7 |

Question 5

| During the past three months, how often have you failed to do what was normally expected of you because of your use of (FIRST DRUG, SECOND DRUG, ETC.) | Never | Once or twice | Monthly | Weekly | Daily or almost daily |
| --- | --- | --- | --- | --- | --- |
| a. Tobacco products (cigarettes, smokes, chewing tobacco, cigars, etc.) |  |  |  |  |  |
| b. Alcoholic beverages (beer, wine, spirits, etc.) | 0 | 5 | 6 | 7 | 8 |
| c. Cannabis (hash, skunk, marijuana, pot, grass, etc.) | 0 | 5 | 6 | 7 | 8 |
| d. Cocaine (coke, crack, charlie, snow etc.) | 0 | 5 | 6 | 7 | 8 |
| e. Amphetamine type stimulants (E, ecstasy, speed, diet pills, etc.) | 0 | 5 | 6 | 7 | 8 |
| f. Inhalants (gas, nitrous, glue, petrol, paint thinner, etc.) | 0 | 5 | 6 | 7 | 8 |
| g. Sedative or sleeping pills (benzodiazepine, rohypnol, zimmos etc.) | 0 | 5 | 6 | 7 | 8 |
| h. Hallucinogens (LSD, acid, mushrooms, PCP, special k, etc.) | 0 | 5 | 6 | 7 | 8 |
| i. Opioids (heroin, morphine, methadone, codeine, etc.) | 0 | 5 | 6 | 7 | 8 |
| j. Headshop products (snowblo, spice, meow meow, BZP, etc.) | 0 | 5 | 6 | 7 | 8 |
| k. Other- specify | 0 | 5 | 6 | 7 | 8 |

***Ask Questions 6 and 7 for all substances ever used (i.e those endorsed in Question 1)***

Question 6

| Has a friend or relative or anyone else ever expressed concern about your use of (FIRST DRUG, SECOND DRUG, ETC.)? | No, never | Yes, in the last 3 months | Yes, but not in the last 3 months |
| --- | --- | --- | --- |
| a. Tobacco products (cigarettes, smokes, chewing tobacco, cigars, etc.) | 0 | 6 | 3 |
| b. Alcoholic beverages (beer, wine, spirits, etc.) | 0 | 6 | 3 |
| c. Cannabis (hash, skunk, marijuana, pot, grass, etc.) | 0 | 6 | 3 |
| d. Cocaine (coke, crack, charlie, snow etc.) | 0 | 6 | 3 |
| e. Amphetamine type stimulants (E, ecstasy, speed, diet pills, etc.) | 0 | 6 | 3 |
| f. Inhalants (gas, nitrous, glue, petrol, paint thinner, etc.) | 0 | 6 | 3 |
| g. Sedative or sleeping pills (benzodiazepine, rohypnol, zimmos etc.) | 0 | 6 | 3 |
| h. Hallucinogens (LSD, acid, mushrooms, PCP, special k, etc.) | 0 | 6 | 3 |
| i. Opioids (heroin, morphine, methadone, codeine, etc.) | 0 | 6 | 3 |
| j. Headshop products (snowblo, spice, meow meow, BZP, etc.) | 0 | 6 | 3 |
| k. Other- specify | 0 | 6 | 3 |

Question 7

| Have you ever tried and failed to control, cut down or stop using (FIRST DRUG, SECOND DRUG, ETC.) | No, never | Yes, in the last 3 months | Yes, but not in the last 3 months |
| --- | --- | --- | --- |
| a. Tobacco products (cigarettes, smokes, chewing tobacco, cigars, etc.) | 0 | 6 | 3 |
| b. Alcoholic beverages (beer, wine, spirits, etc.) | 0 | 6 | 3 |
| c. Cannabis (hash, skunk, marijuana, pot, grass, etc.) | 0 | 6 | 3 |
| d. Cocaine (coke, crack, charlie, snow etc.) | 0 | 6 | 3 |
| e. Amphetamine type stimulants (E, ecstasy, speed, diet pills, etc.) | 0 | 6 | 3 |
| f. Inhalants (gas, nitrous, glue, petrol, paint thinner, etc.) | 0 | 6 | 3 |
| g. Sedative or sleeping pills (benzodiazepine, rohypnol, zimmos etc.) | 0 | 6 | 3 |
| h. Hallucinogens (LSD, acid, mushrooms, PCP, special k, etc.) | 0 | 6 | 3 |
| i. Opioids (heroin, morphine, methadone, codeine, etc.) | 0 | 6 | 3 |
| j. Headshop products (snowblo, spice, meow meow, BZP, etc.) | 0 | 6 | 3 |
| k. Other- specify | 0 | 6 | 3 |

Question 8

| Have you ever used any drug by injection? (NON MEDICAL USE ONLY) | No, never | Yes, in the last 3 months | Yes, but not in the last 3 months |
| --- | --- | --- | --- |
| (Please tick the appropriate box) |  |  |  |

***Patients who have injected drugs in the last 3 months should be asked about their pattern of injecting during this period, to determine their risk levels.***

| **How many times have you injected in the past three months?** |  |
| --- | --- |

**Risk Score Calculator**

*** Do not include Q1 or Q8 in score ***

***Do not calculate a score for tobacco***

| Substance | Q2. Used past 3 months | Q3. Desire/ urge past 3 months | Q4. Social, health, legal, financial problems past 3 months | Q5. Failed Expectations past 3 months | Q6. Friend /Relative concern past 3 months | Q7. Ever tried and failed to cut down | ASSIST Risk Score |
| --- | --- | --- | --- | --- | --- | --- | --- |
| Alcohol |  |  |  |  |  |  |  |
| Cannabis |  |  |  |  |  |  |  |
| Cocaine |  |  |  |  |  |  |  |
| Amphetamines |  |  |  |  |  |  |  |
| Inhalants |  |  |  |  |  |  |  |
| Sedatives |  |  |  |  |  |  |  |
| Hallucinogens |  |  |  |  |  |  |  |
| Opioids |  |  |  |  |  |  |  |
| 'Headshop' products |  |  |  |  |  |  |  |
| Other |  |  |  |  |  |  |  |

**Action Based on ASSIST Risk Scores**

| **Substance** | **Action** | | |
| --- | --- | --- | --- |
|  | **Feedback** | **Feedback and Brief Intervention** | **Brief Intervention and referral** |
| **Alcohol Risk Score** | 0-10 | 11-26 | 27+ |
| **All Other Substances Risk Scores** | 0-3 | 4-26 | 27+ |
| **Intravenous Use** |  | Less than 4 times in the past 3 months | More than 4 times in the past three months |

**Feedback Report Card**

Client name: _______________________________ Date: _________________________

| **Specific Substance Involvement Score** | **Score** | **Risk Level** |
| --- | --- | --- |
| Alcohol |  | 0-10 Low  11-26 Moderate  27+ High |
| Cannabis |  | 0-3 Low  4-26 Moderate  27+ High |
| Cocaine |  | 0-3 Low  4-26 Moderate  27+ High |
| Amphetamine type stimulants |  | 0-3 Low  4-26 Moderate  27+ High |
| Inhalants |  | 0-3 Low  4-26 Moderate  27+ High |
| Sedatives or sleeping pills |  | 0-3 Low  4-26 Moderate  27+ High |
| Hallucinogenic |  | 0-3 Low  4-26 Moderate  27+ High |
| Opioids |  | 0-3 Low  4-26 Moderate  27+ High |
| 'Headshop' products |  | 0-3 Low  4-26 Moderate  27+ High |
| Other- specify |  | 0-3 Low  4-26 Moderate  27+ High |

| **What does your score mean?** |
| --- |
| Low: You have a low risk of problems from your current pattern of use.  Moderate: You have a moderate risk of problems from your current pattern of use.  High: You have a high risk of severe problems from your current pattern of use. You are likely to be dependent. |

| For office use only: ID Number _______________________ |
| --- |

**Screening Tool**

**Date: __________________________ Clinician Name: ____________________________**

**Patient Name: __________________________ Patient dob: ____________________________**

**Action Taken:** No action  Brief Intervention  Brief Intervention and referral

**Introduction:**

- Questions about alcohol and other drug use.
- Across your lifetime and in the last three months.
- Substances can be smoked, snorted, injected or taken in the form of a pill (show response card).
- Prescribed medication will not be recorded.
- Answers are confidential.
- Honest answers are important as we need to know what you actually do.

Question 1

| In your life, which of the following substances have you ever used?  (NON MEDICAL USE ONLY) | No | Yes |
| --- | --- | --- |
| a. Alcoholic beverages (beer, wine, spirits, etc.) | 0 | 3 |
| b. Cannabis (marijuana, pot, grass, hash, etc.) | 0 | 3 |
| c. Cocaine (coke, crack, etc.) | 0 | 3 |
| d. Amphetamine type stimulants (speed, diet pills, ecstasy, etc.) | 0 | 3 |
| e. Inhalants (nitrous, glue, petrol, paint thinner, gas, etc.) | 0 | 3 |
| f. Sedatives or sleeping pills (valium, benzodiazepine, rohypnol, etc.) | 0 | 3 |
| g. Halluncinogens (LSD, acid, mushrooms, PCP, special k, etc.) | 0 | 3 |
| h. Opioids (heroin, morphine, methadone, codeine, etc.) | 0 | 3 |
| i. Headshop' products (snowblo, party pills, go-E, silver bullets, etc.) | 0 | 3 |
| j. Other - specify | 0 | 3 |

Probe if all answers are negative: 'Not even when you were in school?

***If 'No' to all items, stop interview.***

***If 'Yes' to any of these items, ask Question 2 for each substance ever used.***

Question 2

| In the past three months, how often have you used the substances you mentioned (FIRST DRUG, SECOND DRUG, ETC.)? | Never | Once or twice | Monthly | Weekly | Daily or almost daily |
| --- | --- | --- | --- | --- | --- |
| a. Alcoholic beverages (beer, wine, spirits, etc.) | 0 | 2 | 3 | 4 | 6 |
| b. Cannabis (marijuana, pot, grass, hash, etc.) | 0 | 2 | 3 | 4 | 6 |
| c. Cocaine (coke, crack, etc.) | 0 | 2 | 3 | 4 | 6 |
| d. Amphetamine type stimulants (speed, diet pills, ecstasy, etc) | 0 | 2 | 3 | 4 | 6 |
| e. Inhalants (nitrous, glue, petrol, paint thinner, gas, etc.) | 0 | 2 | 3 | 4 | 6 |
| f. Sedatives or sleeping pills (valium, benzodiazepine, rohypnol, etc.) | 0 | 2 | 3 | 4 | 6 |
| g. Halluncinogens (LSD, acid, mushrooms, PCP, special k, etc.) | 0 | 2 | 3 | 4 | 6 |
| h. Opioids (heroin, morphine, methadone, codeine, etc.) | 0 | 2 | 3 | 4 | 6 |
| i. 'Headshop' products (snowblow, party pills, silver bullets, etc.) | 0 | 2 | 3 | 4 | 6 |
| j. Other - specify | 0 | 2 | 3 | 4 | 6 |

***If 'Never' to all items in Question 2, skip to Question 6.***

***If any substance in Question 2 were used in the previous three months, continue with Questions 3, 4, and 5 for each substance used.***

Question 3

| In the past three months, how often have you had a strong desire or urge to use (FIRST DRUG, SECOND DRUG, ETC.)? | Never | Once or twice | Monthly | Weekly | Daily or almost daily |
| --- | --- | --- | --- | --- | --- |
| a. Alcoholic beverages (beer, wine, spirits, etc.) | 0 | 3 | 4 | 5 | 6 |
| b. Cannabis (marijuana, pot, grass, hash, etc.) | 0 | 3 | 4 | 5 | 6 |
| c. Cocaine (coke, crack, etc.) | 0 | 3 | 4 | 5 | 6 |
| d. Amphetamine type stimulants (speed, diet pills, ecstasy, etc.) | 0 | 3 | 4 | 5 | 6 |
| e. Inhalants (nitrous, glue, petrol, paint thinner, gas, etc.) | 0 | 3 | 4 | 5 | 6 |
| f. Sedatives or sleeping pills (valium, benzodiazepine, rohypnol, etc.) | 0 | 3 | 4 | 5 | 6 |
| g. Halluncinogens (LSD, acid, mushrooms, PCP, special k, etc.) | 0 | 3 | 4 | 5 | 6 |
| h. Opiods (heroin, morphine, methadone, codeine, etc.) | 0 | 3 | 4 | 5 | 6 |
| i. 'Headshop' products (snowblow, party pills, silver bullets, etc.) | 0 | 3 | 4 | 5 | 6 |
| j. Other - specify | 0 | 3 | 4 | 5 | 6 |

Question 4

| During the past three months, how often has your use of (FIRST DRUG, SECOND DRUG, ETC.) led to health, social, legal or financial problems? | Never | Once or twice | Monthly | Weekly | Daily or almost daily |
| --- | --- | --- | --- | --- | --- |
| a. Alcoholic beverages (beer, wine, spirits, etc.) | 0 | 4 | 5 | 6 | 7 |
| b. Cannabis (marijuana, pot, grass, hash, etc.) | 0 | 4 | 5 | 6 | 7 |
| c. Cocaine (coke, crack, etc.) | 0 | 4 | 5 | 6 | 7 |
| d. Amphetamine type stimulants (speed, diet pills, ecstasy, etc.) | 0 | 4 | 5 | 6 | 7 |
| e. Inhalants (nitrous, glue, petrol, paint thinner, etc.) | 0 | 4 | 5 | 6 | 7 |
| f. Sedatives or sleeping pills (valium, benzodiazepine, rohypnol, etc.) | 0 | 4 | 5 | 6 | 7 |
| g. Halluncinogens (LSD, acid, mushrooms, PCP, special k, etc.) | 0 | 4 | 5 | 6 | 7 |
| h. Opiods (heroin, morphine, methadone, codeine, etc.) | 0 | 4 | 5 | 6 | 7 |
| i. 'Headshop' products (snowblow, party pills, silver bullets, etc.) | 0 | 4 | 5 | 6 | 7 |
| j. Other - specify | 0 | 4 | 5 | 6 | 7 |

Question 5

| During the past three months, how often have you failed to do what was normally expected of you because of your use of (FIRST DRUG, SECOND DRUG, ETC.)? | Never | Once or twice | Monthly | Weekly | Daily or almost daily |
| --- | --- | --- | --- | --- | --- |
| a. Alcoholic beverages (beer, wine, spirits, etc.) | 0 | 5 | 6 | 7 | 8 |
| b. Cannabis (marijuana, pot, grass, hash, etc.) | 0 | 5 | 6 | 7 | 8 |
| c. Cocaine (coke, crack, etc.) | 0 | 5 | 6 | 7 | 8 |
| d. Amphetamine type stimulants (speed, diet pills, ecstasy, etc.) | 0 | 5 | 6 | 7 | 8 |
| e. Inhalants (nitrous, glue, petrol, paint thinner, etc.) | 0 | 5 | 6 | 7 | 8 |
| f. Sedatives or sleeping pills (valium, benzodiazepine, rohypnol, etc.) | 0 | 5 | 6 | 7 | 8 |
| g. Halluncinogens (LSD, acid, mushrooms, PCP, special k, etc.) | 0 | 5 | 6 | 7 | 8 |
| h. Opiods (heroin, morphine, methadone, codeine, etc.) | 0 | 5 | 6 | 7 | 8 |
| i. 'Headshop' products (snowblow, party pills, silver bullets, etc.) | 0 | 5 | 6 | 7 | 8 |
| j. Other - specify | 0 | 5 | 6 | 7 | 8 |

***Ask Questions 6 and 7 for all substances ever used (i.e those endorsed in Question 1)***

Question 6

| Has a friend or relative or anyone else ever expressed concern about your use of (FIRST DRUG, SECOND DRUG, ETC.)? | No, never | Yes, in the past 3 months | Yes, but not in the past 3 months |
| --- | --- | --- | --- |
| a. Alcoholic beverages (beer, wine, spirits, etc.) | 0 | 6 | 3 |
| b. Cannabis (marijuana, pot, grass, hash, etc.) | 0 | 6 | 3 |
| c. Cocaine (coke, crack, etc.) | 0 | 6 | 3 |
| d. Amphetamine type stimulants (speed, diet pills, ecstasy, etc.) | 0 | 6 | 3 |
| e. Inhalants (nitrous, glue, petrol, paint thinner, etc.) | 0 | 6 | 3 |
| f. Sedatives or sleeping pills (valium, benzodiazepine, rohypnol, etc.) | 0 | 6 | 3 |
| g. Halluncinogens (LSD, acid, mushrooms, PCP, special k, etc.) | 0 | 6 | 3 |
| h. Opiods (heroin, morphine, methadone, codeine, etc.) | 0 | 6 | 3 |
| i. 'Headshop' products (snowblo, party pills, silver bullets, etc.) | 0 | 6 | 3 |
| j. Other - specify | 0 | 6 | 3 |

Question 7

| Have you ever tried and failed to control, cut down or stop using (FIRST DRUG, SECOND DRUG, ETC.)? | No, never | Yes, in the past 3 months | Yes, but not in the past 3 months |
| --- | --- | --- | --- |
| a. Alcoholic beverages (beer, wine, spirits, etc.) | 0 | 6 | 3 |
| b. Cannabis (marijuana, pot, grass, hash, etc.) | 0 | 6 | 3 |
| c. Cocaine (coke, crack, etc.) | 0 | 6 | 3 |
| d. Amphetamine type stimulants (speed, diet pills, ecstasy, etc.) | 0 | 6 | 3 |
| e. Inhalants (nitrous, glue, petrol, paint thinner, etc.) | 0 | 6 | 3 |
| f. Sedatives or sleeping pills (valium, benzodiazepine, rohypnol, etc.) | 0 | 6 | 3 |
| g. Halluncinogens (LSD, acid, mushrooms, PCP, special k, etc.) | 0 | 6 | 3 |
| h. Opiods (heroin, morphine, methadone, codeine, etc.) | 0 | 6 | 3 |
| i. 'Headshop' products (snowblow, party pills, silver bullets, etc.) | 0 | 6 | 3 |
| j. Other - specify | 0 | 6 | 3 |

Question 8

|  | No, never | Yes, in the past 3 months | Yes, but not in the past 3 months |
| --- | --- | --- | --- |
| Have you ever used any drug by injection? (NON MEDICAL USE ONLY) | 0 | 2 | 1 |

Patients who have injected drugs in the last 3 months should be asked about their pattern of injecting during this period, to determine their risk levels and the best course of intervention.

**Risk Score Calculator**

** Do not include Q1 in score.*

| Substance | Q2. Used past 3 months | Q3. Desire/ urge past 3 months | Q4. Social, health, legal, financial problems past 3 months | Q5. Failed Expectations past 3 months | Q6. Friend /Relative concern past 3 months | Q7. Ever tried and failed to cut down | ASSIST Risk Score |
| --- | --- | --- | --- | --- | --- | --- | --- |
| Alcohol |  |  |  |  |  |  |  |
| Cannabis |  |  |  |  |  |  |  |
| Cocaine |  |  |  |  |  |  |  |
| Amphetamines |  |  |  |  |  |  |  |
| Inhalants |  |  |  |  |  |  |  |
| Sedatives |  |  |  |  |  |  |  |
| Hallucinogenics |  |  |  |  |  |  |  |
| Opiods |  |  |  |  |  |  |  |
| 'Headshop' products |  |  |  |  |  |  |  |
| Other |  |  |  |  |  |  |  |

**Action Based on Assist Risk Scores**

| Substance | Feedback | Feedback and Brief Intervention | Brief Intervention and referral to psychiatrist |
| --- | --- | --- | --- |
| **Alcohol** | 0-10 | 11-26 | 27+ |
| **All other substances** | 0-3 | 4-26 | 27+ |
| **Intravenous use** |  | Less than 4 times in the past 3 months | More than 4 times in the past three months |

**Feedback Report Card**

Client name:________________________________ Date: _________________________

| **Specific Substance Involvement Score** | **Score** | **Risk Level** |
| --- | --- | --- |
| Alcohol |  | 0-10 Lower  11-26 Moderate  27+ High |
| Cannabis |  | 0-3 Lower  4-26 Moderate  27+ High |
| Cocaine |  | 0-3 Lower  4-26 Moderate  27+ High |
| Amphetamine type stimulants |  | 0-3 Lower  4-26 Moderate  27+ High |
| Inhalants |  | 0-3 Lower  4-26 Moderate  27+ High |
| Sedatives or sleeping pills |  | 0-3 Lower  4-26 Moderate  27+ High |
| Hallucinogenic |  | 0-3 Lower  4-26 Moderate  27+ High |
| Opiods |  | 0-3 Lower  4-26 Moderate  27+ High |
| 'Headshop' products |  | 0-3 Lower  4-26 Moderate  27+ High |
| Other- specify |  | 0-3 Lower  4-26 Moderate  27+ High |

| **What does your score mean?** |
| --- |
| Lower: You are at a lower risk of health and other problems from your current pattern of use.  Moderate: You are at a moderate risk of health and other problems from your current pattern of substance use.  High: You are at high risk of experiencing severe problems (health, social, financial, legal, relationships) as a result of your current pattern of use and are likely to be dependent. |

**Appendix G: Substance Risk Cards**

**Possible Impact of Alcohol**

(beer, wine, spirits, cider, cans, etc.)

Your risk of having these harms is (tick one):

Low  Moderate  High

| **Regular high use of alcohol is linked with:** | |
| --- | --- |
| Health Problems  Makes Hepatitis worse.  Causes liver damage.  Mental health problems. Such as anxiety, depression and suicide.  Bad impact on how you look. Such as looking older than you are, being over or  under weight or being unfit.  Higher chance of accidents and injury.  Lower sexual performance.  Hangovers, feeling like you need to get sick, getting sick.  Stomach problems.  Problems remembering things and solving  problems, brain damage.  Risk of damage to unborn babies.  Stroke, muscle and nerve damage.  Cancer.  Blackouts and hallucinations.  Higher chance of relapse.  Needing to always drink.  High blood pressure. | Psychosocial Problems  You lose interest in your appearance.  You don't care about anybody else.  Peoples' attitude towards you changes.  Loneliness.  Loss of control of your life.  Children being taken off you.  Money problems. Such as problems paying bills or loans, always borrowing, spending rent on drink, having no money to live on.  Problems with relationships.  Problems in work life.  Problems in family life.  Aggressive and violent actions.  Risky behaviour such as taking sex risks.  Heavy debt.  Difficulty with driving.  Criminal behaviour. Such as being known  to the Gardai, receiving charges or a prison  sentence, shoplifting, pick pocketing. |

| **Alcohol use with methadone is linked to:** |
| --- |
| Can lead to loss of consciousness or death.  Withdrawal symptoms as shortens the time for which methadone is effective.  Loss of take away privileges.  Higher risk of relapse. |

**Possible Impact of Cannabis**

(hash, weed, skunk, blow, shit, dope, marijuana, pot, grass, etc.)

Your risk of having these harms is (tick one):

Low  Moderate  High

| **Regular high use of cannabis is linked with:** | |
| --- | --- |
| Health Problems  Mental health problems. Such as anxiety, panicking, paranoia, psychosis, depression  and suicide.  Bad impact on how you look. Such as looking older than you are, being over or  under weight or being unfit.  Higher chance of accidents and injury.  Lower sexual performance and desire.  Feeling like you need to get sick.  Breathing problems.  Problems paying attention, making  decisions, remembering things and solving  problems.  Slowed reaction time.  Heart disease and high blood pressure.  Cancer.  Needing to use more cannabis and needing  to have cannabis.  Death. | Psychosocial Problems  Loss of motivation.  You lose interest in your appearance.  You don't care about anybody else.  Peoples' attitude towards you changes.  Loneliness.  Loss of control of your life.  Children being taken off you.  Money problems. Such as problems paying bills or loans, always borrowing, spending rent on drugs, having no money to live on.  Problems with relationships.  Problems in work life.  Problems in family life.  Aggressive and violent actions.  Heavy debt.  Difficulty with driving.  Criminal behaviour. Such as being known  to the Gardai, receiving charges or a prison  sentence, shoplifting, pick pocketing,  carrying drugs, buying or selling drugs, sex  work, violent crime, gun crime. |

| **Cannabis use with methadone is linked with:** |
| --- |
| Higher risk of overdose.  Higher risk of relapse. |

**Possible Impact of Cocaine**

(coke, crack, charlie, snow, etc.)

Your risk of having these harms is (tick one):

Low  Moderate  High

| **Regular high use of cocaine is linked with:** | |
| --- | --- |
| Health Problems  Mental health problems. Such as anxiety, psychosis, depression and suicide.  Bad impact on how you look. Such as looking older than you are, being over or  under weight or being unfit.  Numbness, tingling, clammy skin and skin  scratching or picking.  Sleeping problems and tiredness.  Get sick easier.  Lose your voice.  Damage to your nose from snorting.  Problems remembering things.  Injecting cocaine leads to abscesses and  infections as well as Hep C and HIV if  equipment is shared.  Headaches.  Eating problems.  Kidney damage.  Needing to use more cocaine and needing to have cocaine with strong cravings.  Heart disease and high blood pressure. | Psychosocial Problems  You lose interest in your appearance.  You don't care about anybody else.  Peoples' attitude towards you changes.  Loneliness.  Loss of control of your life.  Children being taken off you.  Risky behaviour such as taking sex risks or  sharing needles.  Money problems. Such as problems paying bills or loans, always borrowing, spending rent on drugs, having no money to live on.  Problems with relationships.  Problems in work life.  Problems in family life.  Aggressive and violent actions.  Heavy debt.  Difficulty with driving.  Criminal behaviour. Such as being known  to the Gardai, receiving charges or a prison  sentence, shoplifting, pick pocketing,  carrying drugs, buying or selling drugs, sex  work, violent crime, gun crime. |

| **Cocaine use with methadone is linked with:** |
| --- |
| Loss of take away privileges.  Higher risk of overdose.  Higher risk of relapse.  Changes to your electrocardiogram (ECG) |

**Possible Impact of Amphetamine - Type Stimulants**

(E, ecstasy, speed, diet pills, etc.)

Your risk of having these harms is (tick one):

Low  Moderate  High

| **Regular high use of amphetamines is linked with:** | |
| --- | --- |
| Health Problems  Mental health problems. Such as anxiety, panicking, paranoia, stress, depression and  suicide.  Bad impact on an you are, being over or  under weight or being unfit.  Muscle pain.  Sleeping problems and tiredness.  Eating problems and dehydration.  Jaw clenching.  Headaches.  Get sick easier.  Breathing problems.  Eating problems.  Kidney damage.  Lower sexual performance and desire.  Needing to always take stimulants.  Heart problems and death from a heart  attack.  Problems remembering things.  Liver damage.  Brain haemorrhage.  Psychosis.  Flashbacks. | Psychosocial Problems  You lose interest in your appearance.  You don't care about anybody else.  Peoples' attitude towards you changes.  Loneliness.  Loss of control of your life.  Children being taken off you.  Money problems. Such as problems paying bills or loans, always borrowing, spending rent on drugs, having no money to live on.  Problems with relationships.  Problems in work life.  Problems in family life.  Aggressive and violent actions.  Heavy debt.  Difficulty with driving.  Criminal behaviour. Such as being known  to the Gardai, receiving charges or a prison  sentence, shoplifting, pick pocketing,  carrying drugs, buying or selling drugs, sex  work, violent crime, gun crime. |

| **Amphetamine use with methadone is linked with:** |
| --- |
| Higher risk of overdose.  Higher risk of relapse. |

**Possible Impact of Inhalants**

(gas, nitrous, glue, petrol, paint thinner, etc.)

Your risk of having these harms is (tick one):

Low  Moderate  High

| **Regular high use of inhalants is linked with:** | |
| --- | --- |
| Health Problems  Mental health problems. Such as anxiety, panicking, paranoia, stress, depression and  suicide.  Bad impact on how you look. Such as looking older than you are, being over or  under weight or being unfit.  Flu like symptoms.  Nosebleeds.  Sores around your mouth and nose from  snorting.  Dizziness and hallucinations.  Stomach problems. Such as feeling like you  need to get sick, getting sick and diarrhoea.  Problems remembering things and  confusion.  Tiredness  Organ damage to your heart, lungs, kidney  and liver.  Death from a heart attack.  Slow reactions.  Low oxygen supply. | Psychosocial Problems  You lose interest in your appearance.  You don't care about anybody else.  Peoples' attitude towards you changes.  Loneliness.  Loss of control of your life.  Children being taken off you.  Money problems. Such as problems paying bills or loans, always borrowing, having no  money to live on.  Problems with relationships.  Problems in work life.  Problems in family life.  Aggressive and violent actions.  Heavy debt.  Difficulty with driving.  Criminal behaviour. Such as being known  to the Gardai, receiving charges or a prison  sentence, shoplifting, pick pocketing,  sex work, violent crime, gun crime. |

| **Inhalant use with methadone is linked with:** |
| --- |
| Higher risk of overdose.  Higher risk of relapse. |

**Possible Impact of Sedatives**

(benzodiazepine, rohypnol, zimmos etc.)

Your risk of having these harms is (tick one):

Low  Moderate  High

| **Regular high use of sedatives is linked with:** | |
| --- | --- |
| Health Problems  Mental health problems. Such as anxiety, depression and suicide.  Bad impact on how you look. Such as looking older than you are, being over or  under weight or being unfit.  Dizziness.  Feeling like you need to get sick.  Headaches.  Unsteady balance.  Problems remembering things and paying  attention and confusion.  Tiredness and sleeping problems.  Needing to take more sedatives and needing  to have sedatives quickly. | Psychosocial Problems  You lose interest in your appearance.  You don't care about anybody else.  Peoples' attitude towards you changes.  Loneliness.  Loss of control of your life.  Children being taken off you.  Money problems. Such as problems paying bills or loans, always borrowing, spending rent on drugs, having no money to live on.  Problems with relationships.  Problems in work life.  Problems in family life.  Aggressive and violent actions.  Risky behaviour such as taking sex risks or  sharing needles.  Heavy debt.  Difficulty with driving.  Criminal behaviour. Such as being known  to the Gardai, receiving charges or a prison  sentence, shoplifting, pick pocketing,  carrying drugs, buying or selling drugs, sex  work, violent crime, gun crime. |

| **Sedative use with methadone is linked with:** |
| --- |
| Loss of take away privileges.  Higher risk of overdose.  Higher risk of relapse. |

**Possible Impact of Hallucinogenics**

(LSD, acid, mushrooms, PCP, special k (angel dust, vitamin K, kit kat), ketamine, etc.)

Your risk of having these harms is (tick one):

Low  Moderate  High

| **Regular high use of hallucinogenics is linked with:** | |
| --- | --- |
| Health Problems  Mental health problems. Such as anxiety, depression and suicide.  Bad impact on how you look. Such as looking older than you are, being over or  under weight or being unfit.  Feeling like you need to get sick and getting  sick.  Flashbacks.  Changes your ability to hear, see, smell and  notice changes.  Changes between feeling hot and cold with  sweating.  Tiredness and sleeping problems.  Heart problems. | Psychosocial Problems  You lose interest in your appearance.  You don't care about anybody else.  Peoples' attitude towards you changes.  Loneliness.  Loss of control of your life.  Children being taken off you.  Money problems. Such as problems paying bills or loans, always borrowing, spending rent on drugs, having no money to live on.  Problems with relationships.  Problems in work life.  Problems in family life.  Aggressive and violent actions.  Heavy debt.  Difficulty with driving.  Criminal behaviour. Such as being known  to the Gardai, receiving charges or a prison  sentence, shoplifting, pick pocketing,  carrying drugs, buying or selling drugs, sex  work, violent crime, gun crime. |

| **Hallucinogenic use with methadone is linked with:** |
| --- |
| Higher risk of overdose.  Higher risk of relapse. |

**Possible Impact of Opioids**

(heroin, morphine, methadone, codeine, etc.)

Your risk of having these harms is (tick one):

Low  Moderate  High

| **Regular high use of opioids is linked with:** | |
| --- | --- |
| Health Problems  Mental health problems. Such as anxiety, depression and suicide.  Bad impact on how you look. Such as looking older than you are, being over or  under weight or being unfit.  Itching.  Damage to your teeth.  Lower sexual desire, performance and not  being able to have children.  Tiredness.  Constipation.  Feeling like you need to get sick and getting  sick.  Problems remembering things and paying  attention.  Overdose and death from not breathing.  Needing to take more opioids, needing to  have opioids quickly, strong withdrawal  symptoms. | Psychosocial Problems  You lose interest in your appearance.  You don't care about anybody else.  Peoples' attitude towards you changes.  Loneliness.  Loss of control of your life.  Children being taken off you.  Money problems. Such as problems paying bills or loans, always borrowing, spending rent on drugs, having no money to live on.  Problems with relationships.  Problems in work life.  Problems in family life.  Homelessness.  Aggressive and violent actions.  Heavy debt.  Difficulty with driving.  Criminal behaviour. Such as being known  to the Gardai, receiving charges or a prison  sentence, shoplifting, pick pocketing,  carrying drugs, buying or selling drugs, sex  work, violent crime, gun crime. |

| **Opioid use with methadone is linked with:** |
| --- |
| Loss of take away privileges.  Higher risk of overdose.  Higher risk of relapse. |

**Possible Impact of 'Headshop' Products**

(snowblo, spice, meow meow, BZP, etc.)

Your risk of having these harms is (tick one):

Low  Moderate  High

| **Regular high use of 'headshop' products is linked with:** | |
| --- | --- |
| Health Problems  Mental health problems. Such as anxiety, psychosis, depression and suicide.  Bad impact on how you look. Such as looking older than you are, being over or  under weight or being unfit.  Higher chance of accidents or injury.  Lower sexual performance.  Sleeping problems and tiredness.  Kidney failure.  Smell of head shop products in your sweat.  Confusion and problems remembering  things.  Overdose and loss of consciousness or death  from not breathing.  Injecting head shop products leads to a  higher chance of clots.  Unknown problems as you do not know  what is in the drug.  Seizures.  Heart problems.  Dehydration.  Higher chance of accident and injury.  Feeling 'head-wrecked'.  Risk of damage to unborn babies. | Psychosocial Problems  You lose interest in your appearance.  You don't care about anybody else.  Peoples' attitude towards you changes.  Loneliness.  Loss of control of your life.  Children being taken off you.  Money problems. Such as problems paying bills or loans, always borrowing, spending rent on drugs, having no money to live on.  Problems with relationships.  Problems in work life.  Problems in family life.  Aggressive and violent actions.  Heavy debt.  Difficulty with driving.  Criminal behaviour. Such as being known  to the Gardai, receiving charges or a prison  sentence, shoplifting, pick pocketing,  carrying drugs, buying or selling drugs, sex  work, violent crime, gun crime. |

| **'Headshop' product use with methadone is linked with:** |
| --- |
| Higher risk of overdose.  Higher risk of relapse. |

**Possible Impact of Intravenous Use**

Your risk of having these harms is (tick one):

Low  Moderate  High

| **Intravenous use is associated with:** | |
| --- | --- |
| Health Problems  Higher chance of getting HIV/AIDS and  Hepatitis.  Damage to skin and collapsed veins.  Higher risk of dependence.  Higher risk of mental health problems.  Higher risk of infection, abscesses and  cellulites.  High chance of overdosing.  Scars, bruises, swelling.  Injecting to your neck may lead to a stroke.  DVTs. | Psychosocial Problems  You lose interest in your appearance.  You don't care about anybody else.  Peoples' attitude towards you changes.  Loneliness.  Lose control of your life.  Children being taken off you.  Money problems. Such as problems paying bills or loans, always borrowing, spending rent on drugs, having no money to live on.  Problems with relationships.  Problems in work life.  Problems in family life.  Aggressive and violent actions.  Heavy debt.  Difficulty with driving.  Criminal behaviour. Such as being known  to the Gardai, receiving charges or a prison  sentence, shoplifting, pick pocketing,  carrying drugs, buying or selling drugs, sex  work, violent crime, gun crime. |

| **If you do inject:** |
| --- |
| Always use clean equipment.  Always use a new needle and syringe.  Always use sterile water.  Don't share equipment with others.  Clean the preparation area.  Clean your hands.  Clean the injection site.  Use a different injection site each time  Inject slowly.  Put your used needle in a hard container and dispose of safely. |

**Appendix H: Substance Risk Card Photos**

**Alcohol**


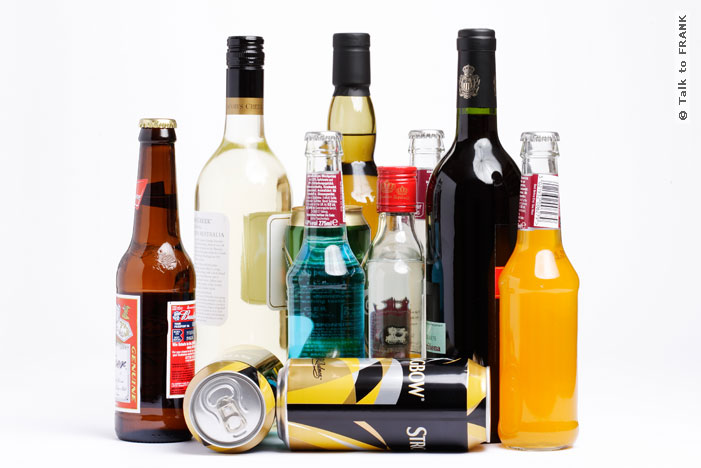


**⇓**


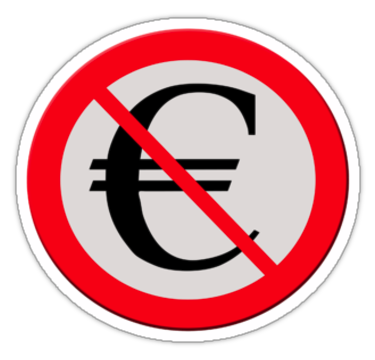

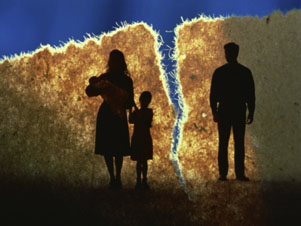

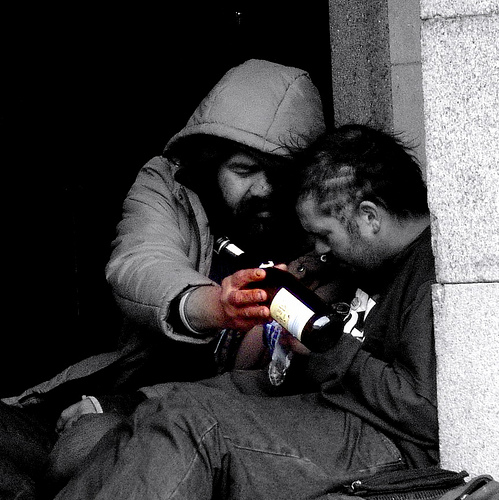


No Money Family Breakdown Homeless


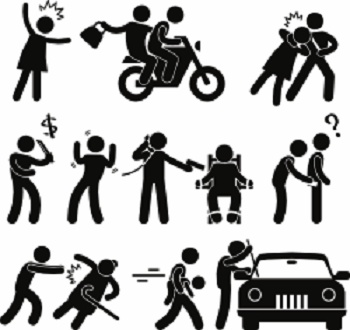

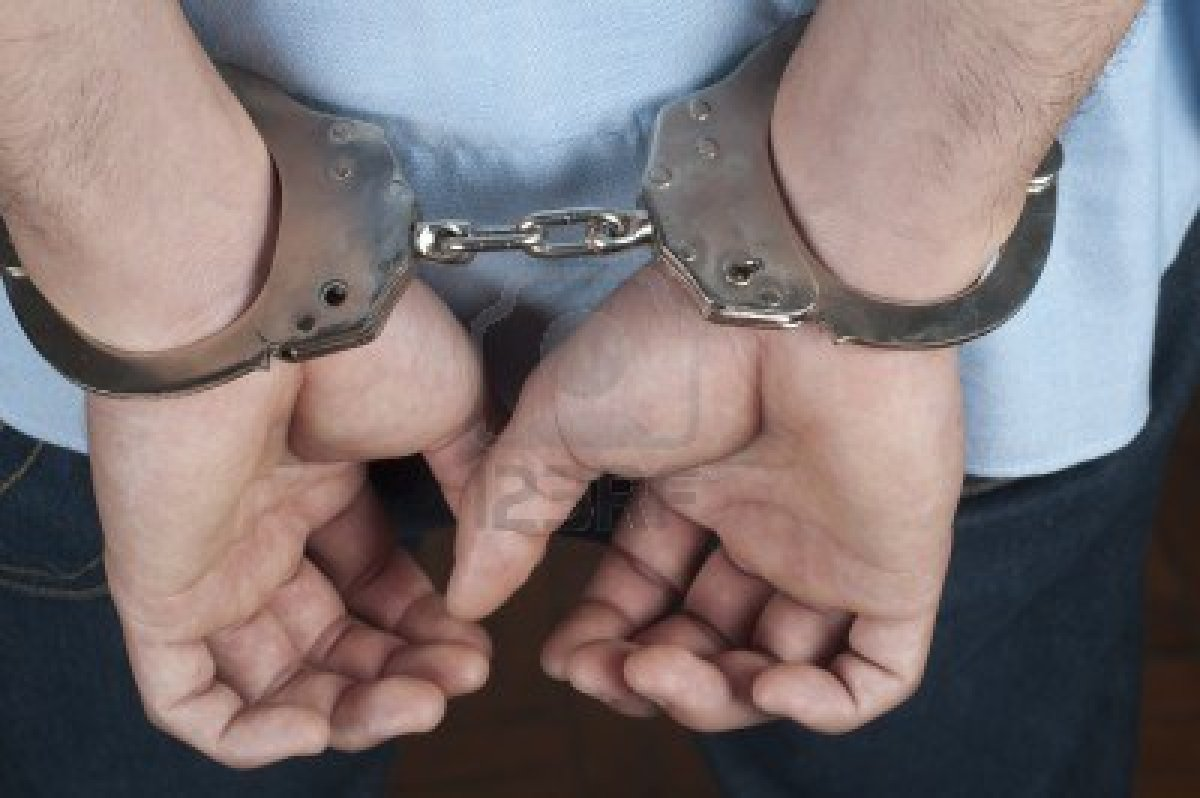

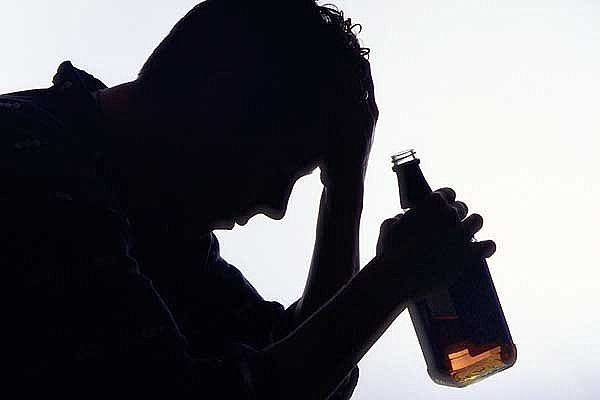


Criminal Behaviour Arrest/Jail Depression


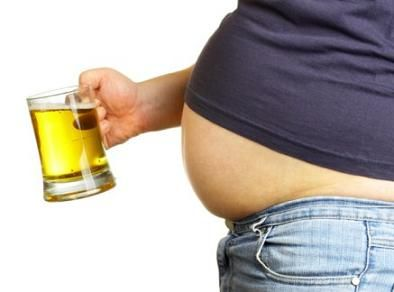

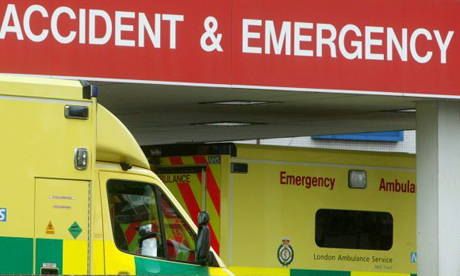


Bad Impact on How You Look Accidents and Injury

**Drugs**


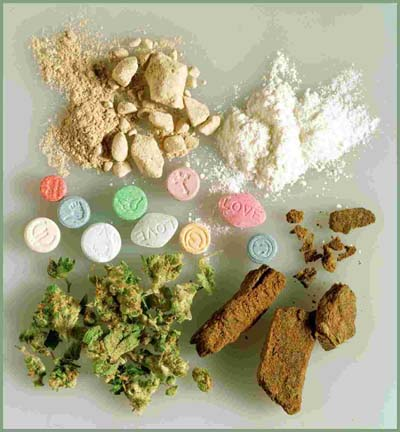


**⇓**


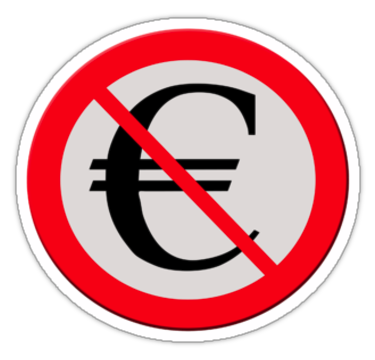

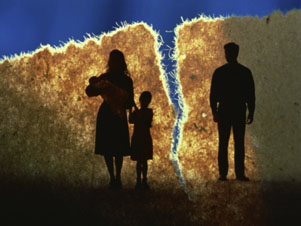

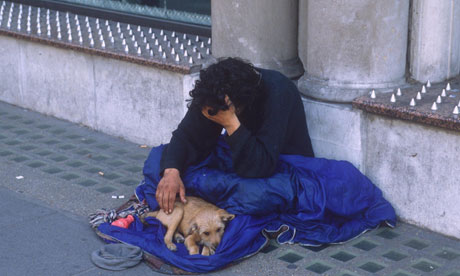


No Money Family Breakdown Homeless


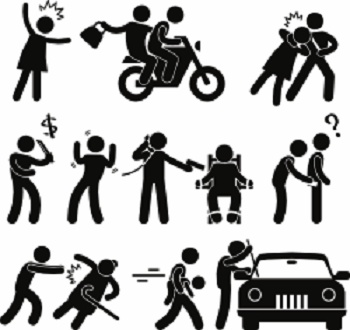

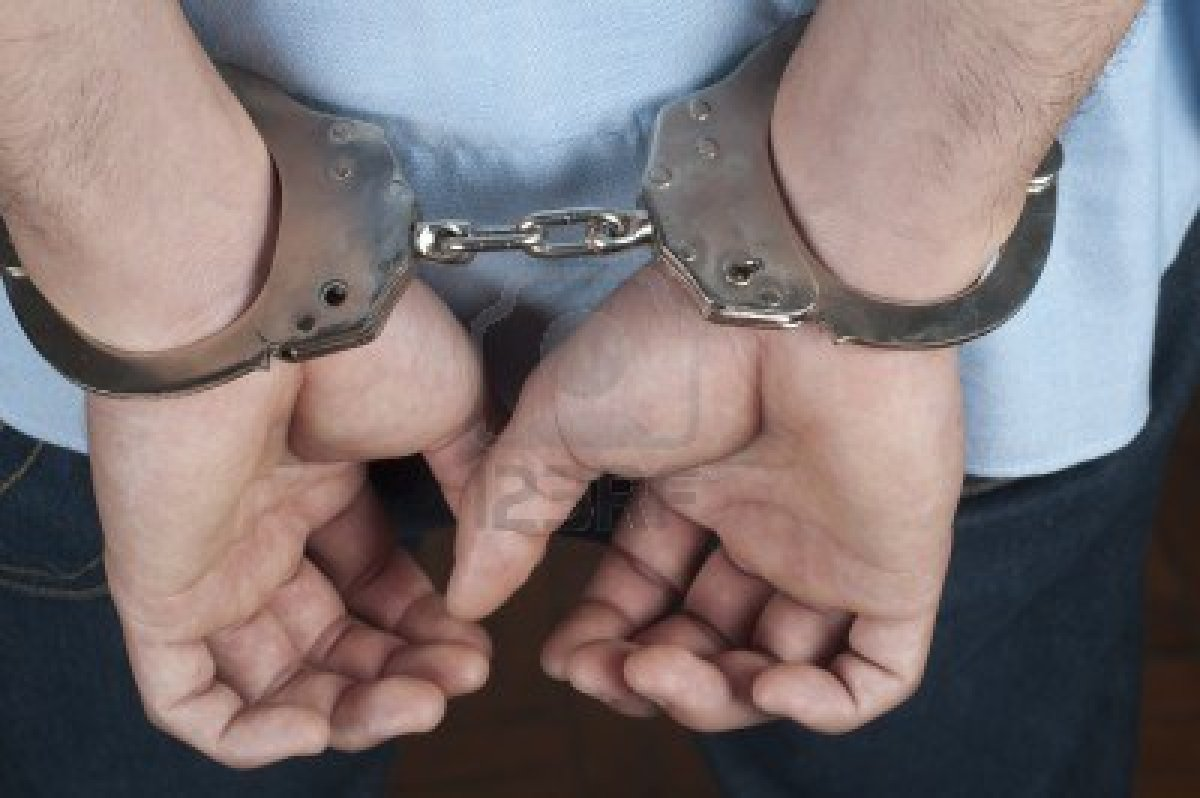

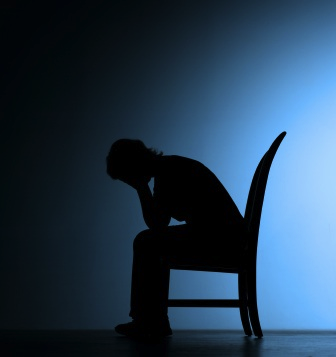


Criminal Behaviour Arrest/Prison Depression


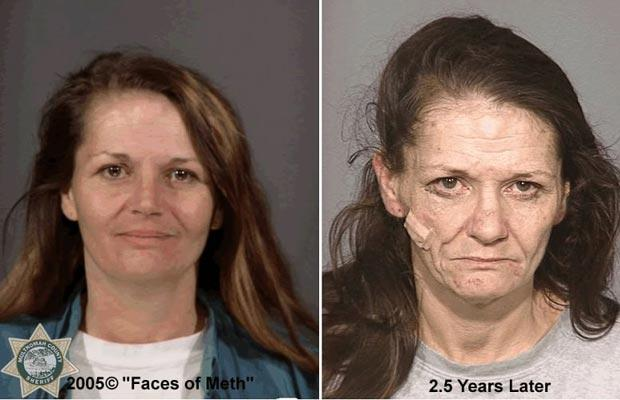

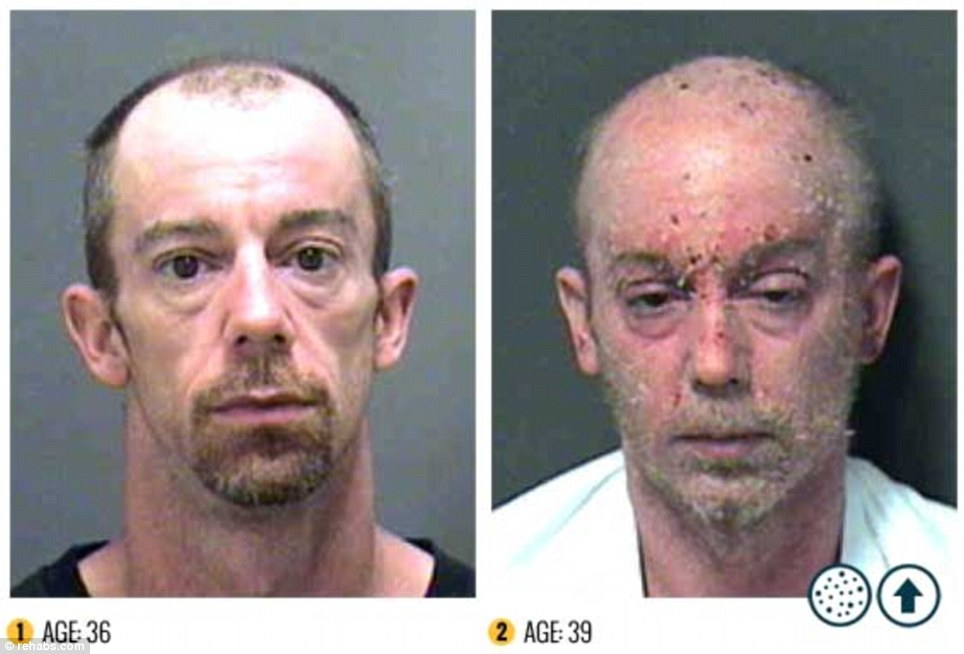


Bad Impact on How You Look

**Injecting**


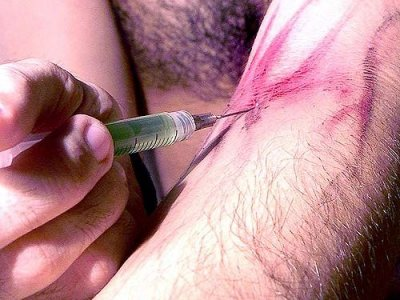


**⇓**

**
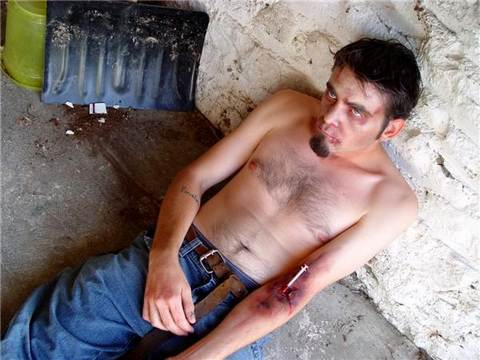
**
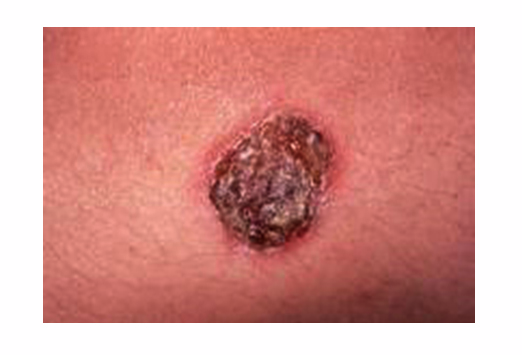


Overdose Infection


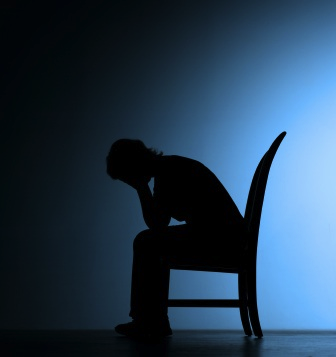

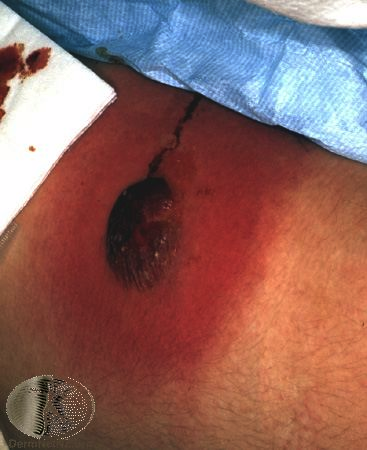


Depression Abscesses


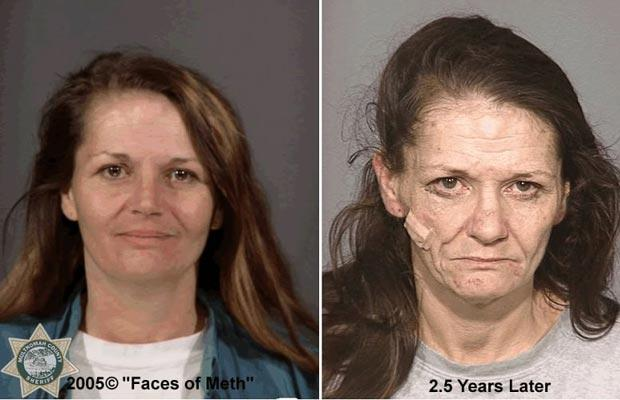

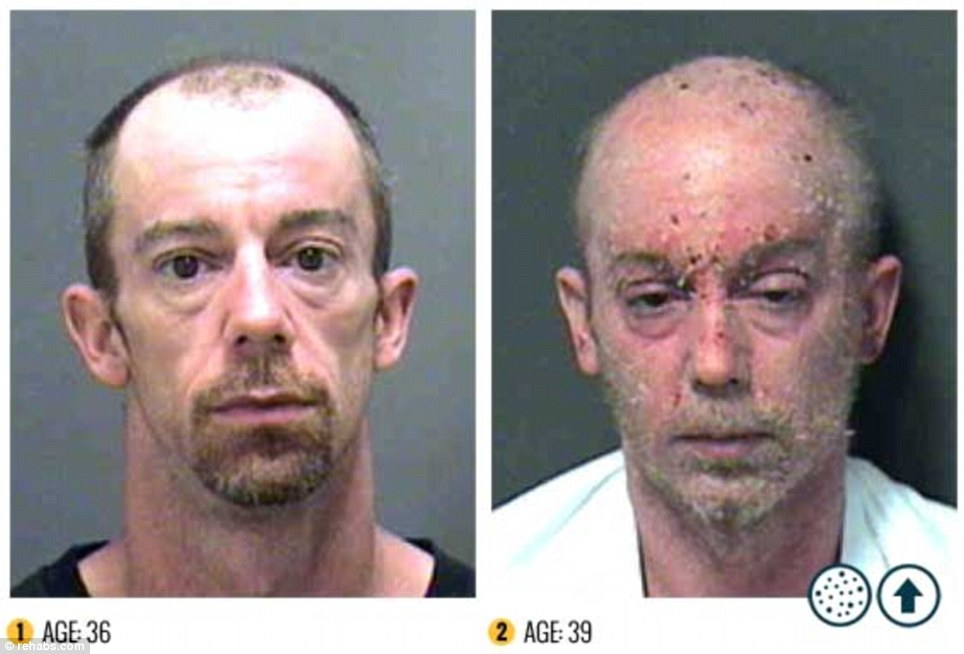


Bad Impact on How You Look

**Appendix I: Readiness to Change Rulers**

**Readiness to Change Rulers**

**'The Readiness Ruler'**

**How important is it for you to stop/cut down your drinking/drug use?**

| 1 | 2 | 3 | 4 | 5 | 6 | 7 | 8 | 9 | 10 |
| --- | --- | --- | --- | --- | --- | --- | --- | --- | --- |
| Not at all Extremely  important important | | | | | | | | | |

**'The Confidence Ruler'**

**How confident are you that you can stop/cut down your drinking/drug use?**

| 1 | 2 | 3 | 4 | 5 | 6 | 7 | 8 | 9 | 10 |
| --- | --- | --- | --- | --- | --- | --- | --- | --- | --- |
| Not at all Extremely  confident confident | | | | | | | | | |

**Appendix J: Pros and Cons For Substance Use Prompts**

**** Only to be used as prompts. Patient suggestions and reasons should be the primary focus****

| Reasons to Quit or Cut Down on Drink/Drugs  *Family and Friends Money*  To prevent family breakdown. To have more money  To avoid children being taken into care. To avoid pay always being gone as soon as you  To have more friends. get it.  Seeing other people get clean. To avoid always borrowing money  Impact of addiction on others. To do a better job at work.  To do a better job at home.  To have better relationships.  *Health Lifestyle*  To live longer and feel better. To have a more normal life.  To sleep better. To be happier.  To be less likely to have a stroke. To avoid doing the same thing day in day out.  To be able to care for yourself for longer. To be treated better by other people.  To reduce the possibility of death from liver disease. To take pride in your appearance.  To prevent problems with medications and methadone. To achieve your goals  To decrease the likelihood of falls or other injuries. Fed up of the addiction lifestyle.  To improve blood pressure control. To have more control over your life.  To reduce the possibility that you will die younger.  To reduce the possibility that you will die in pain.    *Addiction Crime*  To avoid constantly seeking drugs. Upcoming court case.  To decrease the chance of relapse. To avoid being constantly hassled/ questioned/  Previous experience of already being clean. searched by the Gardai.  To avoid being asked if you want drugs all of the time. To avoid committing crimes.  Sick of being looked down upon as a junkie. |
| --- |

| Reasons for Drinking/ Using Drugs  *Feelings Situation*  Because of feelings that nobody cares. Relationship breakdown.  To get out of your head. Habit  To relax or relieve stress. To socialise more easily.  To cope with feelings of anger. Other people expect that you drink with them.  To cope with feelings of boredom. To relieve the stress of arguments with family  To deal with momentary feelings of loneliness. members or friends.  To deal with momentary feelings of depression. For pleasure in social situations.  To deal with feelings of frustration. It's something you do with certain friends or  To make you feel better. relatives.  To relieve pain. Peer pressure.  It's something you do when you smoke.  *Drugs* It's something you do when you watch T.V.  You enjoy the smell, taste or the way it feels. To help you sleep.  To replace another drug. Because it is there.  To help you come down from other substance/drugs. |
| --- |

**Appendix K: Motivational Interviewing**

Motivational Interviewing (MI) is guided by a number of principles. To begin with, discrepancies between actions and goals need to be amplified ^6^. Before any behavioural change occurs there is a period of ambivalence with conflict between actions and goals. The goal of MI is to explore this conflict and to encourage patients to express their reasons for concern and the arguments for change ^9^. Second, clinicians need to roll with resistance reframing, and reflecting reluctance as a new momentum towards change to avoid argumentation and confrontation. Third, clinicians need to express empathy; 'the crucial attitude is respectful listening to the person with a desire to understand his or her perspectives' ^6^. Essential to expressing empathy is acceptance, particularly of patients' ambivalence, which will in turn enable the patient to change. Finally, it is important for clinicians to support patients' self-efficacy. Self-efficacy is a person's belief in his or her capability to carry out and succeed in a specific task. The goals, which patients set, are dependent upon their self-efficacy. Furthermore, self-efficacy also determines whether coping behaviour will be initiated, how much effort will be expended and how long it will be sustained in the face of obstacles and aversive experiences ^36^. Essentially, strong self-efficacy is necessary for change and success. A patient may acknowledge a problem and want to tackle it however, if they do not have strong self-efficacy or perceive any possibility for success then no attempt to change will be made ^7^.

**Appendix L: Screening Led Brief Intervention Example Script**

**Introduction**

**Clinician:** Trinity are doing some research on a new treatment for methadone patients. We will be asking everybody the same questions. Would it be ok for Trinity to use your details in this study? Your details will be kept confidential to the research and treatment teams.

**Patient:** Do I have to?

**Clinician:** You do not have to take part but if you do it is confidential to the treatment and research teams and we will be asking everybody the same questions.

**Patient:** Yeah, I suppose that's ok.

**Consent**

**Clinician:** Good, thank you. Before we start I will get you to sign this consent form. Here is an information sheet about the study so you will know all about the study. Do you understand it?

**Patient:** Yeah.

**Clinician:** Great, you can take it with you and read it again later. The consent form just says that you know what the project is about, that you understand that you do not have to participate, that it is ok for us to ask you the same questions again in three months time and that it is ok for the research team from Trinity to access your records. Don't forget all of your details will be kept safe. Is that all ok?

**Patient:** Yeah, that's grand.

**Clinician:** Great. If you could just sign here, write your name here and then today’s date is the sixth of February 2013 and it is half past one. That is great, I will sign here and then we are ready to get started.

**Ask: Screening Tool**

***Introduction***

**Clinician:** Ok, so, the following questions ask about your experience of using alcohol and other drugs across your lifetime and in the past three months. These substances can be smoked, swallowed, snorted, inhaled or injected. I will give you this sheet here, it has all of the substances that I am going to ask you about and responses to help you to answer the questions. (Show response card).

**Patient:** Thanks, ok.

**Clinician:** For this interview, we will only record substances, which have not been prescribed by a doctor. For example, we will only record street benzos or street methadone, we will not record benzos prescribed by your doctor or the methadone you receive here in the clinic. Does that make sense?

**Patient:** Yeah, that's grand.

***Q1: Lifetime Substance Use***

**Clinician:** Great, so we can get started. In your life have you ever smoked tobacco?

**Patient:** I smoke, yeah.

**Clinician:** Again in your life have you ever drunk alcohol?

**Patient:** Yeah, but not much anymore.

**Clinician:** Have you ever used cannabis?

**Patient:** No.

**Clinician:** So just to check that is hash, weed or pot.

**Patient:** No I never got in to that.

**Clinician:** How about cocaine, have you ever tried cocaine?

**Patient:** I have yeah.

**Clinician:** Ok, and have you tried any kind of amphetamines so that is E or speed?

**Patient:** Yeah, but not for years now.

**Clinician:** What about inhalants, nitrous, gas or glue, anything like that?

**Patient:** Once or twice, but only when I was younger.

**Clinician:** Ok. Have you ever taken sedatives or sleeping pills like benzos?

**Patient:** Yeah.

**Clinician:** How about hallucinogens?

**Patient:** What are they?

**Clinician:** That is LSD, acid or mushrooms.

**Patient:** Oh yeah, I have tried those.

**Clinician:** And opioids?

**Patient:** Well, yeah.

**Clinician:** What about any of the headshop products, snowblo or party pills?

**Patient:** No, I wouldn't touch them, I wouldn't go near that stuff.

**Clinician:** Ok and is there anything else that you can think of that you have tried that is not on that list?

**Patient:** No, I think you have got everything there.

***Q2: Current Substance Use***

**Clinician:** Now I am going to ask you about the substances you have used in the last three months. How often do you smoke?

**Patient:** Every day.

**Clinician:** How often have you dunk alcohol in the last three months?

**Patient:** Just once or twice, twice I'd say.

**Clinician:** How about cocaine, how often have you used cocaine in the last three months?

**Patient:** I would use crack cocaine a couple of times a week, maybe two or three times.

**Clinician:** And what about ecstasy or speed?

**Patient:** I haven't touched anything like that in years.

**Clinician:** And inhalants, have you used any nitrous or gas or glue in the last three months?

**Patient:** No, I only tried that a couple of times years ago.

**Clinician:** What about sedatives, how often have you taken any street benzos or valium or anything like that since Halloween?

**Patient:** I'd take benzos to help me sleep maybe three or four nights a week.

**Clinician:** Ok, and how about Hallucinogens so that was LSD, mushrooms?

**Patient:** No, no, none of that.

**Clinician:** And last one for this question, how often have you used opioids, heroin or methadone outside of the methadone you get here?

**Patient:** A couple of times a week.

***Q3: Craving***

**Clinician:** Still thinking of the last three months, how often do you get a strong craving to smoke?

**Patient:** All the time especially in the morning

**Clinician:** How often have you had a strong desire or urge or craving to drink alcohol?

**Patient:** No, I wouldn't really crave a drink. I don't drink much anymore.

**Clinician:** How about crack, would you get a strong desire or urge for that?

**Patient:** Yeah, I would, I definitely would, everyday I would have a craving for crack.

**Clinician:** What about benzos, would you ever crave them?

**Patient:** No, well no because I have them all of the time so I wouldn't really crave them.

**Clinician:** Ok and how about opioids?

**Patient:** Every day, I want heroin every day.

***Q4: Psychosocial Problems***

**Clinician:** We are nearly half way through now. Again during the last three months, how often has your smoking led to health, social, legal or financial problems?

**Patient:** Well, they are expensive so I'd say I spend a lot of money on them and they don't help my breathing.

**Clinician:** How often would you notice that?

**Patient:** All of the time, everyday.

**Clinician:** How often has your use of alcohol led to health, social, legal or financial problems?

**Patient:** Well I only drank alcohol a couple of times so no it wouldn't cause me any problems.

**Clinician:** What about crack, has that caused you any problems? Has it led to any problems at home or with money or the Gardai or with your physical or mental health? Maybe problems sleeping or concentrating or feeling tired?

**Patient:** Well I would be short of money after buying crack and my boyfriend and my Mam are always at me about using.

**Clinician:** Ok, and how often would you notice those problems?

**Patient:** I don't know, maybe four or five times a week I suppose.

**Clinician:** Alright. Have you noticed any problems from taking benzos?

**Patient:** No, they wouldn't cause me any problems, they help me, they help me sleep.

**Clinician:** What about any impact on your concentration or remembering things or anything like that?

**Patient:** No, no nothing like that.

**Clinician:** Ok, how about the opioids?

**Patient:** Definitely, my breathing, it affects my breathing really bad.

**Clinician:** How often would you notice that?

**Patient:** Well, I would notice that all of the time, so daily.

***Q5: Expectations***

**Clinician:** Ok, in the last three months how often have you failed to do what is normally expected of you because of alcohol?

**Patient:** I, well, what do you mean?

**Clinician:** From drinking alcohol have you missed any appointments, maybe been late to the clinic here? or have you noticed any impact on your any responsibilities, for example, have you forgotten a family birthday?

**Patient:** No, no nothing like that.

**Clinician:** What about crack, have you failed to do what is expected of you from that?

**Patient:** Well, I have missed a few doctors’ appointments.

**Clinician:** How often have you missed those appointments in the last three months?

**Patient:** Only a few times, maybe three or four times.

**Clinician:** And how about benzos, have you failed to do what was expected of you because of that?

**Patient:** Yeah, when I missed the doctor is was because of the benzos as well.

**Clinician:** Ok, and how about opioids, outside of what you get here, has that impacted on your responsibilities?

**Patient:** Yeah, I suppose, I am doing a course and I don't always make it when I am using heroin.

**Clinician:** How often would you miss your course because of that?

**Patient:** I don't know, maybe once a week, around that, once a week.

***Q6: Concern***

**Clinician:** All right, I am going to go back to ask you about your lifetime use again now. Has anybody ever at any stage ever expressed concern about your smoking?

**Patient:** Well the doctor is always telling me I need to give up.

**Clinician:** How about alcohol?

**Patient:** Alcohol has never been a problem for me, so no.

**Clinician:** How about crack, has anybody ever said they were worried about that?

**Patient:** My Mam and my boyfriend are always at me about that.

**Clinician:** So would that be in the last three months?

**Patient:** Yeah, definitely, they are at me all of the time.

**Clinician:** What about E or speed or anything like that?

**Patient:** No, I only did E a few times.

**Clinician:** And how about inhalants so nitrous, gas, glue?

**Patient:** No I was never really into that kind of stuff much.

**Clinician:** Ok has anybody ever expressed concern about your use of benzos or any other sedatives or sleeping pills?

**Patient:** Yeah my Mam and boyfriend are at me about those as well.

**Clinician:** In the last three months?

**Patient:** Yeah.

**Clinician:** How about LSD, acid or mushrooms, has anybody ever spoke to you about that?

**Patient:** No, no.

**Clinician:** What about heroin, has anybody expressed concern about your use of opioids in the last three months?

**Patient:** Well, the doctor is at me in here because of my breathing.

***Q7: Reduction***

**Clinician:** I just have two questions left now. Again thinking of your entire life have you ever tried and failed to cut down, control or stop smoking?

**Patient:** No, I have never tried to give them up.

**Clinician:** What about alcohol?

**Patient:** No, I just kind of started drinking less, other things took over.

**Clinician:** Have you ever tried to cut down or control your use of cocaine or crack?

**Patient:** I have. I went to that Cocaine Anonymous but that was no good, I just went back to using more.

**Clinician:** And was that recently?

**Patient:** Yeah, that was just a month or two ago.

**Clinician:** How about E?

**Patient:** No.

**Clinician:** What about nitrous or gas?

**Patient:** No, I never really had to try and cut down on that, I never got in to that.

**Clinician:** Ok and the benzos have you tried to control those?

**Patient:** I have been trying to stop those since I started going to Cocaine Anonymous.

**Clinician:** And how is that going for you?

**Patient:** They have crept back up. I just need them to help me to relax and sleep.

**Clinician:** What about LSD or mushroom did you ever fail to control those?

**Patient:** No I never had a problem with anything like that.

**Clinician:** And last one, what about opioids have you ever failed to cut down on those?

**Patient:** Yeah, the doctor has been at me about my breathing so I have been trying to stop using as much but it is very hard, so yeah in the last three months.

***Q8: Intravenous Use***

**Clinician:** Ok, so last question Have you ever injected any drugs?

**Patient:** Yes.

**Clinician:** Have you injected in the past three months?

**Patient:** Oh no, no, no I don't do that anymore.

**Clinician:** If you just give me a minute now I will work out your score for you.

**Patient:** Ok.

**Advise**

***Present Screening Results***

**Clinician:** Would you like to see how you scored on the questionnaire you just did?

**Patient:** Yeah.

**Clinician:** You probably know this already but you have scored very high risk for opioids, you scored thirty-eight.

**Patient:** Yeah, I expected that.

**Clinician:** So I am sure you know this but your current pattern of use indicates that you are at a high risk for a number of problems associated with heroin use for your health but also financially and legally. How do you feel about your risk score?

**Patient:** Well I already knew heroin is my biggest problem but I suppose it makes me think about it a bit more.

***Emphasise Individual Responsibility***

**Clinician:** Now obviously any change or decision is entirely up to you. Your substance use is your responsibility. Would you like to talk about possibly changing your substance use? Is that ok?

**Patient:** Yeah.

***Advise Reduction or Abstinence***

**Clinician:** You have already mentioned the impact of your heroin use on your breathing, have you noticed any other impacts from your heroin use?

**Patient:** I think it make me tired sometimes and now this probably sounds stupid but I think it affects my appearance, it makes me look older and it puts a lot of stress on my family and my relationships.

**Clinician:** That is great, they are all consequences of using heroin and very important impacts of using. If you would like to take a look at this card here, it tells you a little bit about the possible consequences of your heroin use and also the impact of using heroin alongside methadone. You probably know most of these side effects already, the ones you have mentioned, your breathing and your appearance are mentioned, but it can be helpful to look at. I am sure you are aware of this, but generally using less heroin leads to a less problems such as those which you have pointed out like the impact on your health and relationships but any decision or change is entirely up to you.

**Patient:** Yeah, I have tried to cut down but it is very hard.

**Assess**

***Patients' Readiness to Change***

**Clinician:** I know it is not easy. Lets see how motivated you are. Look at this sheet here, on a scale of one to ten, where one is not at all important and ten is extremely important, how important do you think it is for you to reduce your use of Heroin?

**Patient:** Maybe about a seven or an eight.

***Weigh up Pros and Cons***

**Clinician:** That is great, really. Why do you want to cut down on your use of heroin?

**Patient:** Well as I said earlier my breathing is really bad and I feel that all of the time. My Mam is also at me about using drugs and I hate always disappointing her. She didn't know I was using for years but then my brother died from injecting Heroin and when my Mam found out I was using she was crushed, she was absolutely devastated, but she is always great to me.

**Clinician:** Sorry to hear about your brother. Are there any other reasons why you want to cut down?

**Patient:** Well, I want a normal life I am sick of not having any money and having to worry about being in trouble with the Gardai. I want to have a normal life, I used to want to be a beautician and have a few kids but that is not going to happen now with the way things are.

***Patients' Readiness to Change***

**Clinician:** They are all very important reasons to change your use of heroin. Your breathing, your Mam and as you say wanting a normal life. Again looking at this sheet on a scale of one to ten, where one is not at all confident and ten is extremely confident, how confident are you that that you can cut down on you heroin use?

**Patient:** It is very difficult, I have tried before and I just can't manage it, maybe a five.

***Weigh up Pros and Cons***

**Clinician:** That is ok, why did you pick a five and not a six?

**Patient:** Like I said I have tried it before and it is so difficult. I have never managed it. I get clean for a few weeks and then I just slip back. You know, I see the people I use with, I bump in to people and they offer me some and I just can't say no. So I just don't know if I can do it.

**Clinician:** I understand you have experience of not managing to stay clean, it is very hard to say no when you are offered exactly what you want. But can I ask, why did you pick a five and not a four or a three?

**Patient:** I want to get clean, I want a normal life and I can manage to get clean it is just staying clean that is the problem.

**Assist**

***Summarise***

**Clinician:** Well you have plenty of good reasons to stay clean your breathing, your Mum and wanting a normal life. And you have experience of trying to cut down before that you feel is against you, but I think you can use that experience to help you succeed this time.

***Negotiate Goals***

**Clinician:** If you were to change your use of Heroin how would you change it?

**Patient:** Well I want to stop, I want to get rid of using Heroin.

**Clinician:** That is a great goal to work towards, you have plenty of your own good reasons to cut down. How about if you were to do it in stages and that would make it a little bit easier for you? What if you were to cut down on the number of times you use a week or the amount you use? How do you think you could go about cutting down?

**Patient:** If it is easier to do it in stages I could use less days of the week.

**Clinician:** So you said you normally use three or four days a week, how about cutting back to two days?

**Patient:** I think I could try that.

**Clinician:** Really, that is great, that would be brilliant if you could cut down to only using two days a week. Can you think of anything you can do to help yourself?

**Patient:** If I can stay away from the people I use with for a while and my Mam and my boyfriend are a great support to me.

**Arrange**

***Refer***

**Clinician:** That is really good, they are great ideas. How about talking to someone in the clinic here? A lot of people find that can be helpful.

**Patient:** If it will help.

**Clinician:** Great well I will give you a referral.

***Take Home Material***

**Clinician:** I will give you this folder to take with you. It has a sheet with your risk score and the Substance Risk Card to remind you of the impact of your heroin use and to help remind you of our conversation.

**Patient:** Thanks

**Clinician:** Thanks for taking part in the study. We will check in with you in three months to see how you are getting on. Best of luck, I believe you can do it if you focus on it.

**Appendix M: References**

1. Humeniuk R, Ali R. Validation of the Alcohol , Smoking and Substance Involvement Screening Test (ASSIST) and Pilot Brief Intervention: A technical report of phase II findings of the WHO ASSIST project. Geneva: World Health Organisation, 2006.

2. Humeniuk R, E., Henry-Edwards S, Ali R, L., Poznyak V, Monteiro M. The Alcohol, Smoking and Substance Involvement Screening Test (ASSIST): manual for use in primary care. 2010.

3. Kumar S, Malhotra A. Brief interventions in substance abuse. Indian J Psychiatry. 2000;42(2):172-83.

4. Henry-Edwards S, Humeniuk R, Ali R, Monteiro M, Poznyak V. Brief Intervention for Substance Use: A Manual for Use in Primary Care. (Draft Version 1.1 for Field Testing). 2003.

5. Prochaska J, O., DiClemente C, C. Towards a comprehensive model of change. In: Miller WR, Heather N, International Conference on Treatment of Addictive B, editors. Treating addictive behaviors : processes of change. New York: Plenum Press; 1986. p. 3-28.

6. Miller WR, Rollnick S. Motivational interviewing : preparing people for change. New York: Guilford Press; 2002.

7. Rollnick S, Allison R. Motivational interviewing. In: Heather N, Stockwell T, editors. The essential handbook of treatment and prevention of alcohol problems. Chichester, West Sussex, England; Hoboken, NJ: J. Wiley; 2004.

8. Dunn C, DeRoo L, Rivara FP. The use of brief interventions adapted from motivational interviewing across behavioral domains: a systematic review. Addiction. 2001;96(12):1725-42.

9. Rollnick S, Heather N, Bell A. Negotiating behaviour change in medical settings: The development of brief motivational interviewing. Journal of Mental Health. 1992;1(1):25-37.

10. Tahan HA, Sminkey PV. Motivational interviewing: building rapport with clients to encourage desirable behavioral and lifestyle changes. Prof Case Manag. 17. United States2012. p. 164-72; quiz 73-4.

11. Anderson R. Living with a Problem Drinker: Your survival guide. Great Britan: Sheldon Press; 2010. 111 p.

12. Humeniuk R, Ali R, Babor T, Souza-Formigoni ML, de Lacerda RB, Ling W, et al. A randomized controlled trial of a brief intervention for illicit drugs linked to the Alcohol, Smoking and Substance Involvement Screening Test (ASSIST) in clients recruited from primary health-care settings in four countries. Addiction. 2012;107(5):957-66.

13. Ali R, Awwad E, Babor T, Bradley F, Butau T, Farrell M, et al. The alcohol, smoking and substance involvement screening test (ASSIST): development, reliability and feasibility. Addiction. 2002.

14. Humeniuk R, Dennington V, Ali R. The Effectiveness of a Brief Intervention for Illicit Drugs Linked to the Alcohol, Smoking and Substance Involvement Screening Test (ASSIST) in Primary Health Care Settings: A technical report of phase III findings of the WHO ASSIST randomised controlled trial. Geneva: World Health Organisation, 2008.

15. Kaner Eileen FS, Dickinson Heather O, Beyer Fiona R, Campbell F, Schlesinger C, Heather N, et al. Effectiveness of brief alcohol interventions in primary care populations. Cochrane Database of Systematic Reviews [Internet]. 2007; (2). Available from: <http://onlinelibrary.wiley.com/doi/10.1002/14651858.CD004148.pub3/abstract>.

16. Babor TF. A Cross-National Trial of Brief Interventions with Heavy Drinkers. American Journal of Public Health. 1996;86(7):948-.

17. Bien T, II, Miller WR, Tonigan JS. Brief interventions for alcohol problems: a review. Addiction. 1993;88(3):315-35.

18. Bennett GA, Edwards S, Bailey J. Helping methadone patients who drink excessively to drink less: short-term outcomes of a pilot motivational intervention. Journal of Substance Use. 2002;7(4):191-7.

19. Darker C, Sweeney B, El Hassan H, Smyth B, Ivers J-H, Barry J. Interventions are effective in reducing alcohol consumption in opiate-dependent methadone-maintained patients: Results from an implementation study. Drug and Alcohol Review. 2012;31:348- 56.

20. Wilk AI, Jensen NM, Havighurst TC. A randomised controlled trial addressing brief interventions in heavy alcohol drinkers. Journal of General Internal Medicine. 1997;12(5):55-64.

21. Baker A, Boggs TG, Lewin TJ. Randomized controlled trial of brief cognitive-behavioural interventions among regular users of amphetamine. Addiction. 2001;96(9):1279-87.

22. Bashir K, King M, Ashworth M. Controlled evaluation of brief intervention by general practitioners to reduce chronic use of benzodiazepines. Br J Gen Pract. 1994;44(386):408-12.

23. Babor TF. Brief Treatments for Cannabis Dependence: Findings From a Randomized Multisite Trial. Journal of Consulting and Clinical Psychology. 2004;72(3):455-66.

24. Copeland J, Swift W, Roffman R, Stephens R. A randomised controlled trial of brief cognitive-behavioural interventions for cannabis use disorder. Journal of Substance Abuse Treatment. 2001;21:55-64.

25. Lang E, Engelander M, Brooke T. Report of an integrated brief intervention with self-defined problem cannabis users. Journal of Substance Abuse Treatment. 2000;19(2):111-6.

26. Bernstein J, Bernstein E, Tassiopoulos K, Heeren T, Levenson S, Hingson R. Brief motivational intervention at a clinic visit reduces cocaine and heroin use. Drug and Alcohol Dependence. 2005;77(1):49-59.

27. Stotts AL, Schmitz JM, Rhoades HM, Grabowski J. Motivational interviewing with cocaine-dependent patients: A pilot study. Journal of Consulting and Clinical Psychology. 2001;69(5):858-62.

28. Saunders B, Wilkinson C. The impact of a brief motivational intervention with opiate users attending a methadone programme. Addiction. 1995;90(3):415-24.

29. Sorsdahl K, Stein J, Dan., Weich L, Fourie D, Myers B. The effectiveness of a hospital-based intervention for patients with substance-use problems in the Western Cape. South African Medical Journal. 2012;102(7):634-5.

30. McCambridge J, Strang J. RESEARCH REPORT The efficacy of single-session motivational interviewing in reducing drug consumption and perceptions of drug-related risk and harm among young people: results from a multi-site cluster randomized trial. Addiction. 2004;99(1):39-52.

31. Pal HR, Yadav D, Mehta S, Mohan I. A comparison of brief intervention versus simple advice for alcohol use disorders in a North India community-based sample followed for 3 months. Alcohol and Alcoholism. 2007;42(4):328-32.

32. Martin G, Copeland J. Brief intervention for regular ecstasy (MDMA) users: Pilot randomized trial of a Check-up model. Journal of Substance Use. 2010;15(2):131-42.

33. Heather N, Bowie A, Ashton H, McAvoy B, Spencer I, Brodie J, et al. Randomised controlled trial of two brief interventions against long-term benzodiazepine use: outcome of intervention. Addiction Research & Theory. 2004;12(2):141-54.

34. Kahan M, Wilson L, Becker L. Effectiveness of physician-based interventions with problem drinkers: a review. Canadian Medical Association Journal. 1995;152(6):851-9.

35. Moyer A, Finney JW, Swearingen CE, Vergun P. Brief interventions for alcohol problems: a meta-analytic review of controlled investigations in treatment-seeking and non-treatment-seeking populations. Addiction. 2002;97(3):279-92.

36. Bandura A. Self-efficacy: Toward a unifying theory of behavioral change. Psychological Review. 1977;84(2):191-215.
